# The ubiquitin-editing enzyme A20 (*TNFAIP3*): mechanisms of activation, biological function, diseases and therapeutic targets

**DOI:** 10.1186/s43556-026-00557-7

**Published:** 2026-09-07

**Authors:** Junying Hu, Xingchen Liu, Wenqiao Zhou, Bing Zhong, Feng Liu

**Affiliations:** Department of Otolaryngology-Head and Neck Surgery, West China Hospital of Sichuan University, Chengdu, Sichuan China

**Keywords:** A20, *TNFAIP3*, Ubiquitin editing, Inflammation, Cancer, Therapeutic targeting

## Abstract

A20, encoded by tumor necrosis factor alpha-induced protein 3 (*TNFAIP3*), is a ubiquitin-editing enzyme that acts as a central regulator of inflammatory signal termination, immune homeostasis and tissue protection. By controlling receptor-proximal ubiquitin signaling, A20 limits the amplitude and duration of inflammatory responses and thereby restrains the transition from acute host defense to chronic tissue-damaging inflammation. Genetic, biochemical and disease-model studies have linked altered A20 expression or function to a broad spectrum of human disorders, including monogenic autoinflammation, autoimmunity, barrier and fibrotic diseases, infection and cancer. However, the literature remains fragmented across organ systems and recurrent signaling pathways, obscuring the conserved biological functions of A20 and the contexts in which these functions become pathogenic. Here, we examine the mechanisms governing A20 activation, recruitment and ubiquitin-editing logic, with emphasis on the distinct contributions of the ovarian tumor (OTU) domain and zinc-finger (ZnF) modules. The major biological functions of A20 are then organized into recurrent output modules, including immune-cell homeostasis, signal termination, coordination of cell-death programmes, and oxidative-stress or immunometabolic adaptation. Disease associations are discussed according to dominant pathological outputs and vulnerable cellular compartments rather than by repetitive pathway listing. Finally, the therapeutic implications of A20 are assessed, including its potential as a biomarker and context-dependent therapeutic node, together with the translational challenges posed by cell-type specificity, disease-stage dependence and immune risk. A20 emerges as a context-dependent regulator of inflammation, tissue remodeling and tumour progression with relevance for biomarker development and precision therapeutic targeting.

## Introduction

A20 is a ubiquitin-editing enzyme encoded by tumor necrosis factor alpha-induced protein 3 (*TNFAIP3*) and is a central regulator of inflammatory signal termination, immune homeostasis, and tissue protection [1–5]. Through coordinated control of ubiquitin-dependent signaling, A20 limits the amplitude and duration of receptor-proximal inflammatory responses and thereby restricts the transition from acute host defense to chronic tissue-damaging inflammation [1–3]. Over the past two decades, genetic, biochemical, and disease-model studies have established A20 as a key checkpoint at the interface of innate and adaptive immunity, cell-death control, and stress adaptation [1, 6, 7]. Consistent with this broad regulatory role, altered A20 expression or function has been implicated in monogenic autoinflammatory syndromes, autoimmune disease, chronic tissue remodeling, infection, and multiple cancers [1, 7–10].

Despite this progress, the rapidly expanding literature has remained difficult to integrate. A20-associated disease studies are often organized either by individual organ systems or by recurrent signaling pathways, with the result that similar mechanistic themes are repeatedly described across disorders without a unifying framework. This is particularly evident for pathways such as nuclear factor kappa B (NF-κB), inflammatory cell death, and stress-associated adaptation, which recur across diseases but are expressed through different tissue compartments and pathological outputs [1, 4–6, 11]. The same difficulty is apparent in cancer, where A20 may appear tumour suppressive in some contexts yet tumour promoting in others [12, 13]. A synthesis that distinguishes conserved A20 functions from their disease-specific manifestations is therefore needed in order to define more clearly how this ubiquitin-editing checkpoint contributes to human pathology.

This Review considers A20 from four connected perspectives. First, the mechanisms that govern A20 activation, recruitment, and ubiquitin-editing logic are examined, with particular attention to the distinct contributions of the ovarian tumor (OTU) domain and zinc-finger (ZnF) modules. Second, the major biological functions of A20 are organized into recurrent output modules, including immune-cell homeostasis, signal termination, coordination of cell-death programmes, and oxidative stress or immunometabolic adaptation. Third, disease associations are discussed according to their dominant pathological outputs rather than by repetitive pathway listing, with emphasis on the cellular compartment in which defective A20 restraint becomes clinically relevant. Finally, the therapeutic implications of A20 are assessed, including the opportunities and limitations of targeting a molecule whose function is both central and strongly context dependent. By integrating molecular mechanism, biological function, disease expression, and translational potential, this Review aims to provide a more coherent framework for understanding how A20 shapes human disease.

## Mechanisms of A20 activation and regulation

The biological effects of A20 arise from the integration of its modular protein structure, inducible expression, receptor-proximal recruitment and selective recognition of ubiquitin-modified signaling complexes. Rather than functioning solely as a deubiquitinase, A20 acts as a multifunctional ubiquitin-editing and scaffold protein whose output depends on the engaged receptor, ubiquitin-chain topology and cellular context. This section therefore examines the structural features, activation mechanisms and regulatory layers that provide the molecular basis for the immune, cell-death and stress-adaptive functions discussed in the following section.

### Domain architecture and ubiquitin-chain recognition

**Fig. 1:**
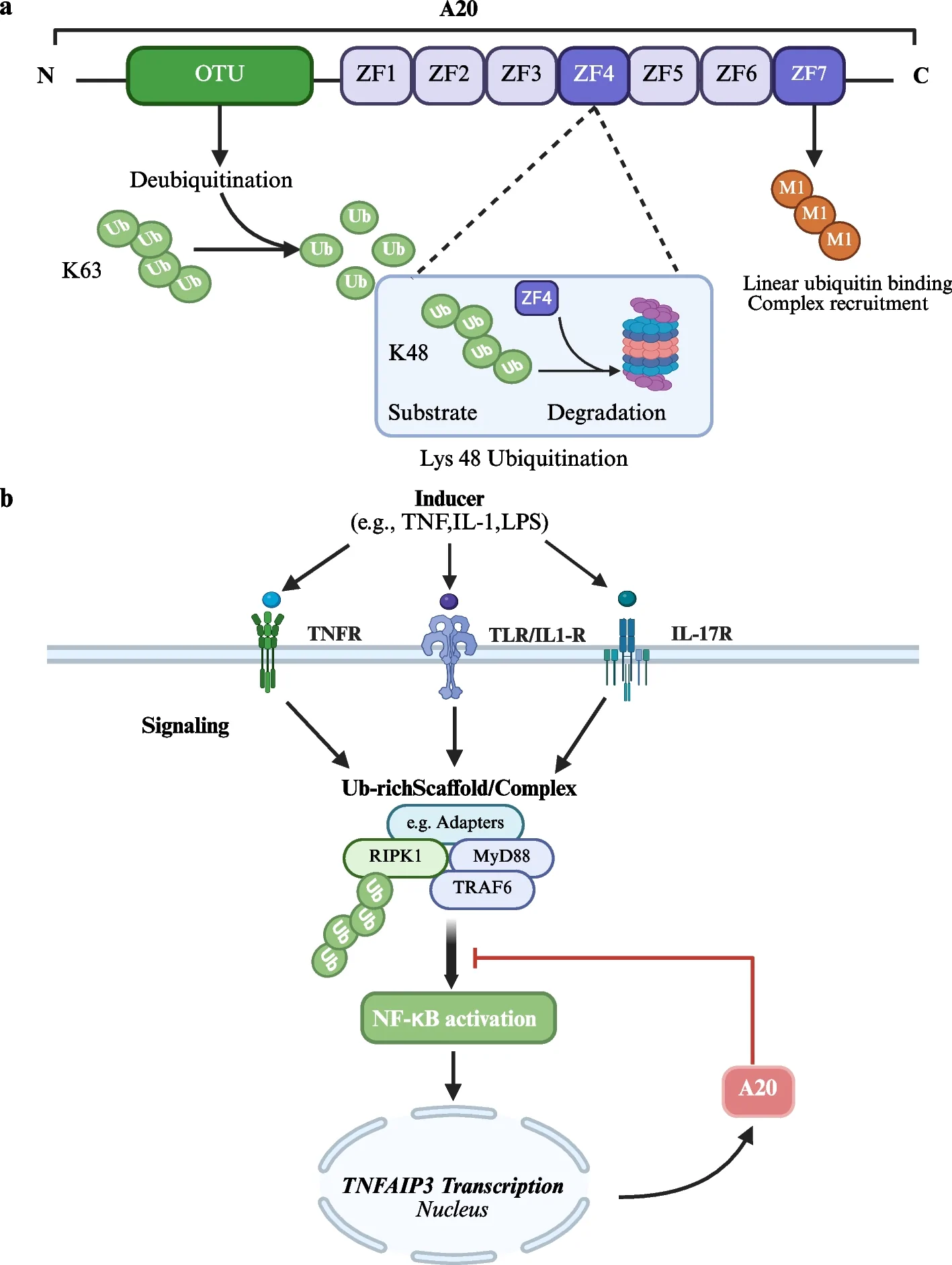
Domain architecture of A20 and its induction at receptor-proximal signaling complexes. **a** A20 contains an N-terminal OTU deubiquitinase domain and seven C-terminal ZnF motifs. The OTU domain is shown removing K63-linked ubiquitin chains. ZnF4 is shown in association with K48-linked ubiquitination and proteasomal degradation of selected substrates, whereas ZnF7 is shown binding linear (M1-linked) ubiquitin chains and facilitating recruitment to ubiquitin-rich signaling complexes. **b** Pro-inflammatory stimuli, including tumor necrosis factor (TNF), interleukin-1 (IL-1) and lipopolysaccharide (LPS), activate receptor systems such as TNFR, TLR/IL-1R and IL-17R, leading to the formation of ubiquitin-rich signaling scaffolds containing adaptor proteins such as RIPK1, MyD88 and TRAF6. Downstream NF-κB activation induces TNFAIP3 transcription, and the resulting A20 protein feeds back to limit signaling intensity and duration. Red blunt-ended lines indicate inhibitory effects; black arrows indicate activation or directional influence. The figure summarizes representative mechanisms underlying A20-mediated ubiquitin editing and inflammatory signal regulation. Created with BioRender.com. Abbreviations: A20, tumor necrosis factor alpha-induced protein 3; OTU, ovarian tumor domain; ZnF, zinc-finger; K63, lysine 63-linked ubiquitin chain; K48, lysine 48-linked ubiquitin chain; M1, methionine 1-linked (linear) ubiquitin chain; TNF, tumor necrosis factor; IL-1, interleukin-1; LPS, lipopolysaccharide; TNFR, tumor necrosis factor receptor; TLR, Toll-like receptor; IL-1R, interleukin-1 receptor; IL-17R, interleukin-17 receptor; RIPK1, receptor-interacting serine/threonine-protein kinase 1; MyD88, myeloid differentiation primary response 88; TRAF6, TNF receptor-associated factor 6; NF-κB, nuclear factor kappa B; TNFAIP3, tumor necrosis factor alpha-induced protein 3

A20, encoded by *TNFAIP3*, is a cytoplasmic ubiquitin-editing enzyme composed of an N-terminal OTU deubiquitinase domain and seven C-terminal ZnF motifs [9, 14, 15]. This modular organization provides the structural basis for chain-type–aware ubiquitin editing within receptor-proximal signaling complexes (Fig. 1). Current evidence most strongly supports a division of labor in which the OTU domain mediates hydrolytic deubiquitination, whereas specific ZnF modules contribute to ubiquitin binding, complex recruitment and, in some settings, ubiquitin-ligase–associated outputs [7, 14, 16]. The OTU domain functions as a deubiquitinase that preferentially hydrolyses K63-linked ubiquitin chains, with reduced activity toward K11- and K48-linked chains, thereby attenuating signalling through the removal of activating ubiquitin modifications from key intermediates such as RIP1 [7, 14]. Among the ZnF motifs, ZnF4 and ZnF7 are the best characterized. ZnF4 has been linked to K48-linked ubiquitination and proteasomal turnover of selected substrates, whereas ZnF7 binds linear (M1-linked) ubiquitin chains and facilitates recruitment of A20 to ubiquitin-rich receptor complexes. This framework is supported by structural and functional studies summarized in recent reviews and is currently one of the more secure aspects of A20 biology. By contrast, the relative contribution of the remaining ZnF motifs to disease-specific biology remains less completely resolved [9, 14, 16]. The most widely cited example of coordinated domain function is the regulation of receptor-interacting serine/threonine-protein kinase 1 (RIPK1)-associated tumor necrosis factor receptor 1 (TNFR1) signaling, in which A20 has been described to remove activating ubiquitin chains and promote subsequent proteolytic inactivation [17, 18]. Although this general logic is well established, the precise sequence and domain dependence of these editing events may differ across systems [7].

### Induction and feedback control at receptor-proximal complexes

A20 functions predominantly at receptor-proximal signaling platforms, where ubiquitin scaffolds organize kinase activation and downstream transcriptional responses [19, 20] (Fig. 1). In this setting, A20 acts as an inducible negative-feedback regulator that is recruited after inflammatory stimulation to limit the amplitude and duration of signaling [4, 5, 21]. In TNFR1 signaling, A20 has been shown to interact with RIPK1-associated complexes and to reduce tumor necrosis factor (TNF)-driven NF-κB activation [14, 22]. In Toll-like receptor (TLR)/interleukin-1 receptor (IL-1R) pathways, signaling proceeds through myeloid differentiation primary response 88 (MyD88), interleukin-1 receptor-associated kinase (IRAK) and TNF receptor-associated factor 6 (TRAF6), and A20 has been implicated in restraining TRAF6-associated ubiquitin signaling, thereby dampening pathway activity [14, 23, 24]. These mechanisms are among the best-supported examples of receptor-proximal A20 action. However, the degree to which identical logic applies across all receptors should not be overstated. A20 also intersects with additional receptor cascades, including interleukin-17 receptor (IL-17R)-associated signaling, and may constrain mitogen-activated protein kinase (MAPK) outputs in selected immune contexts [4, 25–27]. Here the evidence is biologically convincing but mechanistically more heterogeneous. In most systems, the literature supports a model in which A20 limits c-Jun N-terminal kinase (JNK)- or MAPK-associated inflammatory outputs through upstream ubiquitin-scaffold remodeling, rather than through a fully resolved direct MAPK substrate mechanism [4, 28]. Together, inducibility and feedback control position A20 as a context-tuned molecular brake that prevents prolonged receptor-proximal signaling from evolving into chronic, tissue-damaging inflammation [5, 28, 29]. This conclusion is well supported at the systems level, although the receptor-specific molecular details remain unevenly defined [7, 25, 30].

### Transcriptional induction and activity tuning

A defining feature of A20 biology is that it is rapidly inducible by inflammatory cues [14]. Pro-inflammatory stimuli such as TNF, IL-1 and lipopolysaccharide (LPS) induce TNFAIP3 transcription through NF-κB, thereby establishing a canonical negative-feedback loop [31, 32]. This inducible behavior is central to the idea that A20 does not simply constitutively suppress inflammation, but is actively mobilized when receptor signaling reaches a threshold that threatens tissue homeostasis [2, 7, 33–35].

Inflammatory signaling may also enhance A20 function after induction. In particular, phosphorylation of Ser381 has been reported to increase A20 inhibitory activity [15, 36–38]. Thus, A20 regulation occurs at least two separable levels: transcriptional induction, which determines availability, and post-induction tuning, which influences how effectively A20 can restrain inflammatory signaling once expressed [16, 39–41]. This distinction is useful when interpreting disease states in which TNFAIP3 transcription is intact but A20 function nevertheless appears insufficient [7, 9, 42–44].

### Post-transcriptional regulation

Beyond transcriptional induction, A20 abundance is shaped by post-transcriptional regulatory networks, particularly those involving microRNAs, long non-coding RNAs and, in some contexts, circular RNAs [16, 45–48]. Many of these regulators act through the 3′ untranslated region (3′UTR) of TNFAIP3 mRNA or through competing endogenous RNA circuits, thereby influencing mRNA stability or effective translation [49–51]. This layer of regulation is increasingly relevant in inflammatory disease, where altered RNA networks can either facilitate timely A20 accumulation or suppress A20 expression and thereby weaken feedback control [46, 49, 51]. At present, the biological importance of these RNA circuits is well supported by multiple disease-focused studies, but mechanistic interpretation requires caution [45, 47, 52, 53]. In many cases, the evidence rests on expression analyses, luciferase/targeting assays, and gain- or loss-of-function studies in cultured cells [52]. Such data can strongly support a regulatory interaction, but they do not always establish how broadly a given RNA–A20 axis operates across cell types or disease stages [7, 9]. Therefore, mechanistic synthesis should specify the cellular compartment in which a given RNA regulator acts, because epithelial, myeloid, lymphoid and endothelial contexts may produce distinct phenotypic consequences [46, 54, 55].

### Post-translational regulation and subcellular routing

Post-translational control provides an additional regulatory layer over A20 function [7, 16]. As noted above, phosphorylation can alter inhibitory capacity, but A20 abundance and activity are also likely to be influenced by protein turnover, subcellular localization, and the handling of A20-associated signaling intermediates [11, 43, 56, 57]. In broad terms, these processes affect not only how much A20 is available, but also whether A20 is present in the appropriate molecular context to edit its substrates [42, 57]. Compared with receptor-proximal ubiquitin editing, this layer of biology is less uniformly resolved [7, 9, 58].

### Substrate selection and context determinants: a testable framework

A recurring challenge in the field is to explain how A20 selects substrates and why its net effect appears protective in some settings yet maladaptive in others [6, 9, 11, 42, 59]. Current evidence can be organized into a conceptual framework with three interdependent levels: availability, recruitment, and editing mode [9, 42, 43]. Although this framework does not fully resolve substrate selection across all systems, it provides a useful basis for interpreting the context dependence of A20 biology.

The first level, availability, refers to whether sufficient functional A20 is present in a given cellular context. This depends on transcriptional induction, post-transcriptional regulation, and post-translational tuning of A20 activity [10, 16, 43]. The second level, recruitment, refers to whether A20 is engaged at the relevant signaling platform. Because A20 acts predominantly within receptor-proximal and stress-responsive complexes, its biological effect depends not only on abundance but also on access to the appropriate ubiquitin-rich scaffold [9, 26, 42]. The third level, editing mode, refers to the dominant output once A20 is recruited: in some settings, catalytic chain removal mediated by the OTU domain is likely to be most relevant, whereas in others, ZnF-dependent ubiquitin binding, complex engagement, or turnover-associated functions may predominate [11, 42, 43].

This framework helps explain why A20 deficiency or dysfunction can lead to divergent pathological consequences. In settings characterized by persistent receptor-proximal inflammatory signaling, impaired A20 availability or recruitment is expected to prolong NF-κB-centred cytokine programs and exacerbate tissue injury [3, 5, 59]. By contrast, in established tumours or stressed tissues, altered recruitment or a shift in editing mode may favour stress tolerance, survival, or immune evasion [9, 13, 60]. The biological outcome therefore reflects not a single intrinsic property of A20, but the interaction between cellular compartment, stimulus type and duration, ubiquitin-chain environment, and the broader stress state of the cell [8].

## Biological functions of A20

A20 exerts its biological effects through interconnected functional modules rather than through a single linear pathway. In immune cells, it calibrates activation thresholds, preserves tolerance and shapes compartment-specific effector programmes [1, 7]. At the signalling level, A20 terminates receptor-proximal inflammatory cascades, most prominently NF-κB-centred signalling, while also intersecting with MAPK, Janus kinase–signal transducer and activator of transcription (JAK–STAT), and phosphoinositide 3-kinase–protein kinase B–mechanistic target of rapamycin (PI3K–AKT–mTOR) pathways in a context-dependent manner [7, 14, 44]. Beyond signal termination, A20 influences the balance between inflammatory activation, regulated cell death, autophagy, oxidative-stress control and immunometabolic adaptation [61–63]. This section therefore organizes the biological functions of A20 into four major output modules: immune-cell homeostasis and tolerance, signal termination, coordination of cell-death and stress-adaptation programmes, and oxidative-stress or immunometabolic adaptation.

### Immune-cell homeostasis and tolerance

A20 is a central regulator of immune-cell homeostasis across both innate and adaptive compartments [1, 7]. Rather than functioning as a generic suppressor of inflammation, A20 calibrates activation thresholds, survival programmes and effector outputs in a cell-type-dependent manner [4, 6, 40, 54]. This interpretation is supported most strongly by studies in defined immune-cell populations, including macrophages, dendritic cells, T cells and B cells, together with disease models in which cell-restricted A20 perturbation produces distinct immune phenotypes [14, 64] (Table 1).

**Table 1:** A20-associated functions across immune-cell compartments

Immune-cell compartment	Dominant A20-associated role	Representative outputs of reduced or altered A20 function
Macrophages/myeloid cells	Restraint of inflammatory activation, immune polarization and necroptotic cell fate	STAT1-dependent IFN-γ skewing, impaired type 2 immunity, Th1/Th17-biased inflammation, NLRP3 activation and RIPK1–RIPK3–MLKL-dependent necroptosis
CD4⁺ T cells	Control of activation, mTOR/autophagy balance and survival fitness	Increased mTOR activity, reduced autophagy-dependent survival and enhanced Th1/Th2/Th17 responses
Treg cells	Regulation of Treg development and plasticity	Altered NF-κB dynamics, reduced IL-2 dependence and impaired suppression of IL-17A-associated plasticity
Th17-related responses	Restriction of pathogenic effector programmes	Enhanced Th17-skewed inflammation and IL-22-driven effector activity
B cells	Checkpoint of B-cell activation and humoral tolerance	B-cell hyperresponsiveness, germinal-centre expansion, autoantibody production, immune-complex deposition and enhanced NF-κB/STAT1/mTOR signalling

*A20* tumor necrosis factor alpha-induced protein 3, *STAT1* signal transducer and activator of transcription 1, *IFN-γ* interferon gamma, *Th1* T helper type 1 cell, *Th2* T helper type 2 cell, *Th17* T helper 17 cell, *NLRP3* NOD-like receptor family pyrin domain-containing 3, *RIPK1* receptor-interacting serine/threonine-protein kinase 1, *RIPK3* receptor-interacting serine/threonine-protein kinase 3, *MLKL* mixed lineage kinase domain-like protein, *CD4* cluster of differentiation 4, *Treg* regulatory T cell, *mTOR* mechanistic target of rapamycin, *NF-κB* nuclear factor kappa B, *IL-2* interleukin-2, *IL-17A* interleukin-17A, *IL-22* interleukin-22

In the myeloid compartment, cell-restricted knockout models, inflammatory disease models and ex vivo macrophage studies identify A20 as a central brake on inflammatory activation while preserving tissue-protective immune programmes [4, 6, 40, 54, 65, 66]. In vivo, myeloid A20 deficiency redirects IL-33-driven lung immunity from ILC2/eosinophilic responses toward a STAT1-dependent, IFN-γ-associated programme, and during helminth infection similarly impairs alternative macrophage polarization and type 2 immunity while favouring Th1/Th17-skewed inflammation, consistent with a context-dependent role in calibrating macrophage-driven immune polarization [6, 54]. In DSS colitis, A20 likewise limits macrophage inflammatory output through NF-κB-associated pathways, although the evidence in that setting is primarily functional rather than substrate-resolved [67]. Mechanistically, the level of support differs across pathways: IL-33 studies define a myeloid A20–STAT1 axis without identifying a direct biochemical target, whereas inflammasome control is supported by direct evidence that A20 binds NEK7, promotes its K48-linked ubiquitination and proteasomal degradation, and thereby limits NLRP3 activation [54, 68]. A20 also restrains inflammatory cell fate, as genetic studies show that it suppresses RIPK1–RIPK3–MLKL-dependent macrophage necroptosis through its ZnF7 ubiquitin-binding domain, thereby preventing secondary inflammasome-driven pathology [69].

Within T-cell compartments, A20 acts as a context-dependent brake on activation, survival and effector polarization, but the strength of evidence differs across subsets. In CD4⁺ T cells, conditional mouse genetics and ex vivo stimulation studies show that A20 restrains mTOR activity and promotes autophagy-dependent survival after T-cell receptor ligation, supporting a cell-intrinsic role in maintaining activated T-cell fitness [29]. Functional studies in allergic and autoimmune inflammation further indicate that reduced A20 expression is associated with enhanced Th1-, Th2- and Th17-skewed responses, although these findings derive mainly from disease models and knockdown systems rather than direct molecular target assignment [70, 71]. In Treg compartments, more mechanistic resolution is available: T cell-specific A20 deficiency increases thymic Treg development in a cell-intrinsic manner and is associated with altered NF-κB dynamics and reduced IL-2 dependence [72], whereas in human effector Treg, TNF–TNFR2–A20 signalling suppresses IL-17A expression and limits Treg plasticity, with kinome analyses implicating TCR-, JAK- and MAPK-associated pathways [73]. At the level of pathogenic Th17 responses, the strongest domain-resolved evidence shows that A20 restrains IL-22-driven effector programmes through its ZnF7 ubiquitin-binding motif [42].

**Fig. 2:**
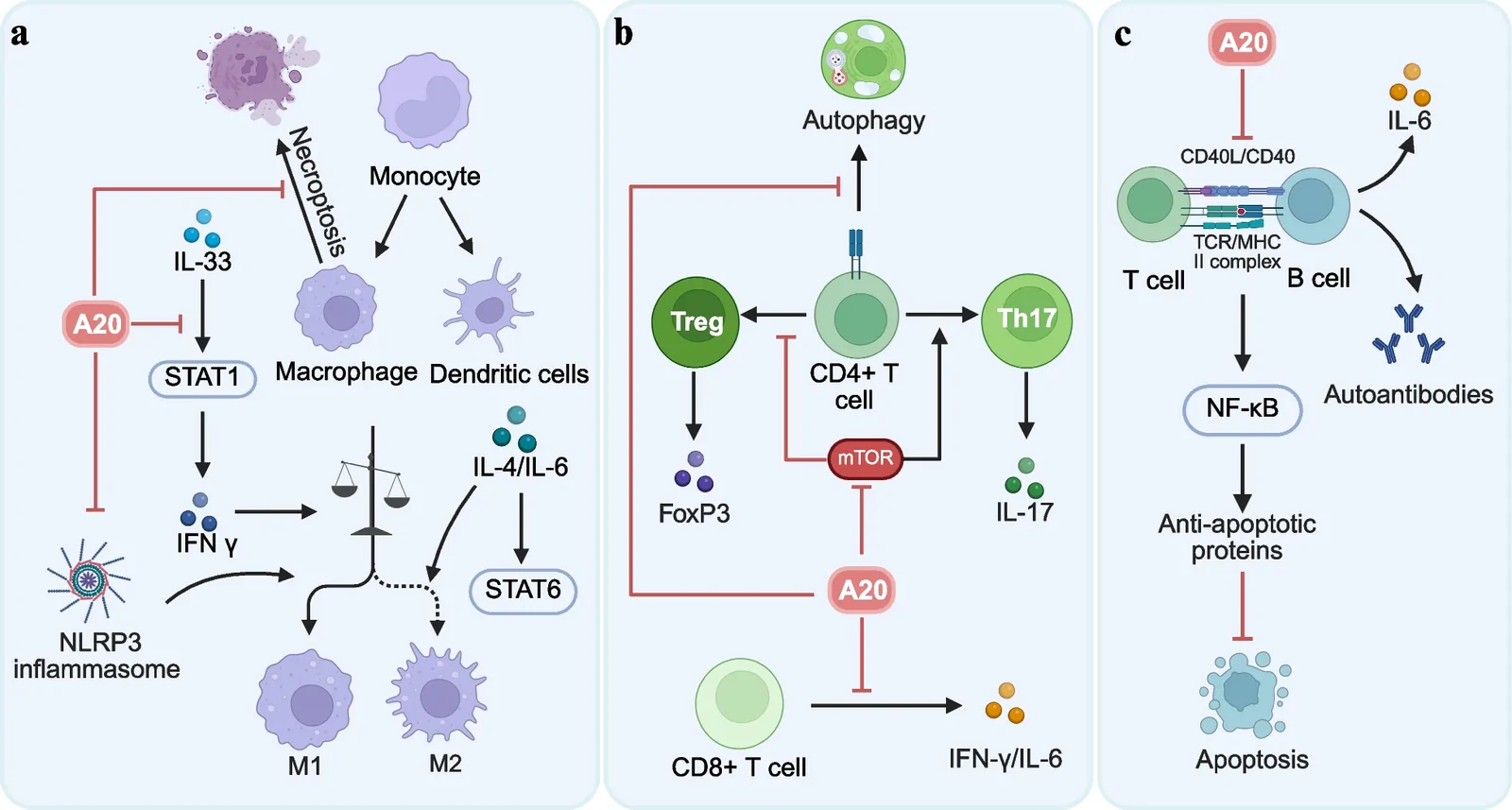
A20 in immune-cell homeostasis and tolerance. **a** A20 in myeloid-cell compartments. The scheme illustrates A20-associated restraint of inflammatory myeloid outputs, including necroptosis, NLRP3 inflammasome activity and IL-33–STAT1–IFN-γ-linked signaling, together with effects on macrophage polarization. Additional myeloid populations, including monocytes and dendritic cells, are shown to indicate the broader myeloid context.** b** A20 in T-cell compartments. In CD4⁺ T cells, A20 is shown in association with mTOR restraint, autophagy and the balance between FoxP3⁺ regulatory T-cell and T helper 17 (Th17) programs. In CD8⁺ T cells, A20 is shown limiting effector cytokine output.** c** A20 in B-cell tolerance. The scheme depicts A20 in relation to T-cell-dependent B-cell activation, NF-κB-associated survival signaling and autoantibody production. Red blunt-ended lines indicate inhibitory effects; black arrows indicate activation or directional influence. The figure summarizes representative immune-cell outputs associated with A20. Created with BioRender.com. Abbreviations: A20, tumor necrosis factor alpha-induced protein 3; NLRP3, NOD-like receptor family pyrin domain-containing 3; IL-33, interleukin-33; STAT1, signal transducer and activator of transcription 1; IFN-γ, interferon gamma; M1, classically activated macrophage; M2, alternatively activated macrophage; CD4, cluster of differentiation 4; CD8, cluster of differentiation 8; Treg, regulatory T cell; FoxP3, forkhead box P3; mTOR, mechanistic target of rapamycin; Th17, T helper 17 cell; IL-17, interleukin-17; IL-6, interleukin-6; NF-κB, nuclear factor kappa B; TCR, T-cell receptor; MHC, major histocompatibility complex

In B cells, recent studies position A20 as a quantitative gatekeeper of activation and tolerance rather than a simple brake on inflammation. Using B cell-specific genetic perturbation models, Diehl, et al. showed that A20 defines a narrow window of permissible B-cell reactivity, such that modest hyperreactivity promotes autoimmunity whereas stronger hyperreactivity can instead provoke cytotoxic T-cell-mediated elimination, revealing a non-linear checkpoint that limits both autoinflammation and lymphomagenesis [66]. Human genetic and functional data further link reduced A20 activity to enhanced NF-κB-, STAT1- and mTOR-associated signalling, although these findings derive from mixed PBMC populations and therefore do not directly resolve B-cell-intrinsic mechanisms [44]. These more recent observations build on earlier B cell-specific conditional knockout studies showing that A20 deficiency drives germinal-centre expansion, autoantibody production and immune-complex deposition, and that purified A20-deficient B cells are hyperresponsive to BCR-, CD40- and TLR-mediated stimulation. Mechanistically, the strongest direct evidence still supports a model in which A20 restrains CD40-driven canonical and non-canonical NF-κB signalling, thereby limiting Bcl-x-dependent survival and preserving susceptibility to Fas-mediated deletion of activated B cells [74]. Collectively, these studies identify A20 as a central regulator of humoral tolerance whose principal function is to set the threshold at which activated B cells are either contained, deleted or allowed to become pathogenic (Fig. 2).

### Signal termination modules

**Fig. 3:**
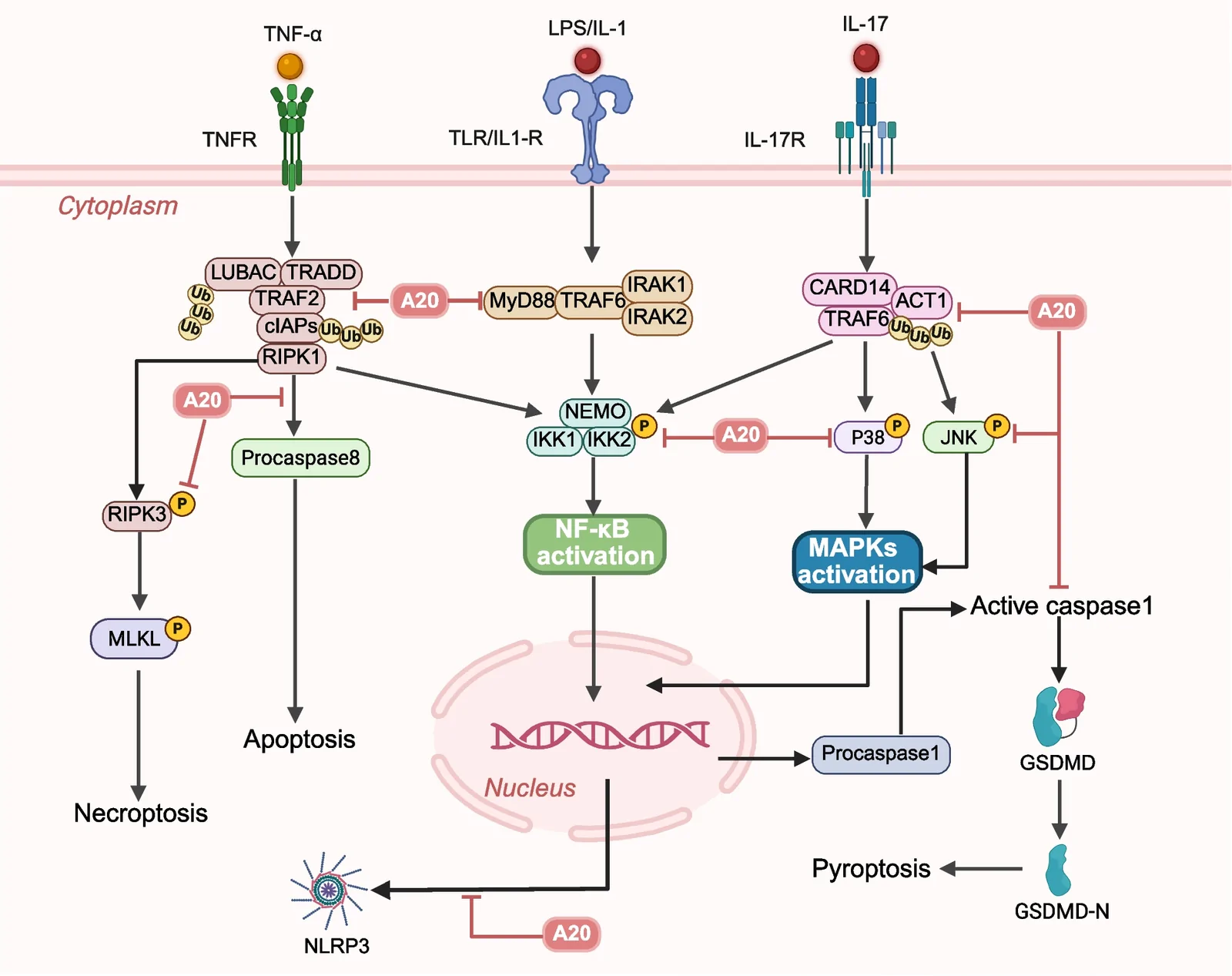
A20-associated signaling modules linking receptor-proximal inflammatory signaling to cell-death outputs. The scheme depicts representative A20-regulated signaling modules downstream of tumour necrosis factor receptor (TNFR), Toll-like receptor/interleukin-1 receptor (TLR/IL-1R) and interleukin-17 receptor (IL-17R). Receptor-proximal complexes containing intermediates such as RIPK1, MyD88, TRAF6, IRAK1/2, ACT1 and CARD14 are shown in relation to NF-κB and MAPK activation, as well as apoptosis, necroptosis and pyroptosis-associated outputs. A20 is shown at multiple positions within these modules to indicate its established role in limiting signaling intensity and shaping downstream inflammatory and cell-death responses. Red blunt-ended lines indicate inhibitory effects of A20; black arrows indicate activation or directional progression; circled P indicates phosphorylation; ubiquitin symbols indicate ubiquitin-modified intermediates. The figure summarizes representative pathway relationships discussed in the text and does not imply equivalent mechanistic resolution for all depicted interactions. Created with BioRender.com. Red blunt-ended lines indicate inhibitory effects of A20; black arrows indicate activation or directional progression; circled P indicates phosphorylation; ubiquitin symbols indicate ubiquitin-modified intermediates. Abbreviations: A20, Tumor necrosis factor alpha-induced protein 3; TNF-α, Tumor necrosis factor alpha; TNFR, Tumor necrosis factor receptor; IL-1, Interleukin-1; LPS, Lipopolysaccharide; TLR, Toll-like receptor; IL-1R, Interleukin-1 receptor; IL-17R, Interleukin-17 receptor; RIPK1, Receptor-interacting serine/threonine-protein kinase 1; MyD88, Myeloid differentiation primary response 88; TRAF6, TNF receptor-associated factor 6; IRAK1/2, Interleukin-1 receptor-associated kinase 1/2; ACT1, NF-κB activator 1; CARD14, Caspase recruitment domain-containing protein 14; NF-κB, Nuclear factor kappa B; MAPK, Mitogen-activated protein kinase; NLRP3, NOD-like receptor family pyrin domain-containing 3; MLKL, Mixed lineage kinase domain-like protein; GSDMD, Gasdermin D; Ub, Ubiquitin

Having discussed the molecular basis of A20 recruitment and ubiquitin editing above, this subsection considers signal termination as a biological output of A20 activity. Across receptor systems, A20 controls the intensity, duration and downstream consequences of inflammatory signalling rather than acting through an identical substrate mechanism in every context (Fig. 3). Taken together, A20 is supported most strongly as a core regulator of NF-κB-centred receptor-proximal signalling, whereas its effects on MAPK, JAK–STAT, and PI3K–AKT–mTOR pathways appear more context dependent and are supported more often by functional perturbation and disease-model associations [7, 14, 44].

#### NF-κB

NF-κB is the best-defined signalling output restrained by A20 [7, 14]. Functionally, A20 limits the magnitude and duration of NF-κB activation downstream of TNFR, TLR and IL-1R stimulation, thereby restricting cytokine production, inflammatory survival programmes and tissue-remodelling responses [42]. This function reflects A20-dependent editing of receptor-proximal ubiquitin scaffolds, as discussed above, but its biological significance extends beyond the receptor complex to determine whether inflammatory transcription resolves or persists. Among A20-regulated pathways, NF-κB therefore provides the clearest link between molecular ubiquitin editing and tissue-level inflammatory control [9].

#### MAPK

MAPK signalling is also restrained by A20, although the mechanism is less uniform than for NF-κB and is best defined at the level of upstream ubiquitin scaffolds rather than MAPK kinases themselves. The clearest direct evidence comes from noncanonical TGF-β signalling, in which A20 is recruited to TRAF6 via Smad6 and removes K63-linked ubiquitin chains, thereby limiting downstream TAK1- and p38/JNK-dependent signalling; this conclusion is supported by ubiquitination assays, knockdown experiments and in vivo analyses [75]. Consistent with this model, TNFR1 complex-level studies show that A20 regulates K63- and M1-linked ubiquitin networks required for TAK1 recruitment, thereby indirectly constraining downstream MAPK outputs [76]. In endothelial cells, A20 overexpression or silencing modulates TAK1 and p38 phosphorylation, apoptosis and eNOS signalling, but these data derive mainly from cell-based perturbation systems and do not identify direct ubiquitin substrates [77]. Additional inflammatory models further associate reduced A20 with enhanced JNK/p38/ERK activation, although these findings remain largely pathway-level and correlative [78]. Thus, current evidence supports a receptor-context-specific model in which A20 limits MAPK signalling primarily by editing TRAF6–TAK1-associated ubiquitin platforms, with evidence strength ranging from direct ubiquitin-dependent mechanisms to functional disease-model observations.

#### JAK/STAT

JAK–STAT signalling is increasingly recognized as an additional pathway constrained by A20, although the supporting evidence is less uniform than for NF-κB and is strongest for STAT1 rather than for the pathway as a whole. The clearest mechanistic evidence comes from myeloid-specific A20-deficient mice and derived macrophages, in which A20 loss increases STAT1 mRNA and protein abundance, enhances STAT1-dependent gene transcription, and promotes enthesitis that is ameliorated by JAK inhibition; importantly, this effect is selective, as comparable regulation of STAT3 was not consistently observed in the same system [79]. Consistent with this, cell-based knockdown studies in plasmacytoid dendritic cells show that A20 silencing enhances STAT1 phosphorylation and augments maturation, cytokine production and migration, indicating that A20 limits not only STAT1 expression but also STAT1-dependent functional activation in defined immune-cell contexts [80]. More recent patient-derived PBMC analyses also link reduced A20 function to enhanced STAT1 activation, although these data derive from mixed immune-cell populations and therefore do not define cell-intrinsic targets with the same precision [44]. By contrast, evidence from leukemia and psoriasis-associated systems mainly supports a functional or correlative relationship between A20 and JAK/STAT signalling, rather than a shared substrate-level mechanism [81–83]. Taken together, current evidence supports a model in which A20 restrains JAK–STAT output primarily through suppression of STAT1 expression and activation, but, unlike NF-κB, no universal direct biochemical mechanism has yet been established.

#### PI3K/AKT/mTOR

Beyond its canonical effects on NF-κB, A20 also shapes PI3K–AKT–mTOR signalling, albeit with substantially less mechanistic uniformity. Here, the strongest evidence converges on mTOR-associated complexes and their downstream effects on autophagy and cell survival, rather than on a single pathway-wide mechanism. In activated CD4⁺ T cells, Tnfaip3 deficiency augments mTOR activity, diminishes autophagy and compromises survival; notably, A20 associates with the mTOR complex, and its loss is accompanied by increased ubiquitination of this complex, supporting a relatively direct role in constraining mTOR-dependent signalling in this setting [63]. A related, though more context-restricted, mechanism has been described in hepatocellular carcinoma cells, in which A20 interacts with mTORC2 and limits the mTORC2–Akt–Rac1 module [84]. By comparison, evidence for regulation of upstream PI3K–Akt output remains less resolved. In vascular smooth muscle cells, A20 overexpression suppresses TNF-induced PI3K/Akt/GSK3β/CREB signalling and limits proliferation and migration, but the relevant ubiquitin substrates were not defined [84]. Functional support for this axis also comes from inflammatory nucleus pulposus cells and HA20 patient-derived PBMCs, where altered A20 expression or function is associated with mTOR-linked changes in autophagy and inflammatory output; however, these observations derive from perturbation systems or mixed-cell populations and therefore do not establish substrate-resolved mechanisms [44, 85]. Taken together, current data favour a context-dependent model in which A20 restrains PI3K–AKT–mTOR signalling primarily through modulation of mTOR-associated complexes and their downstream programmes, while a universal direct biochemical mechanism for the pathway as a whole remains to be established.

### Coordination of cell-death and stress-adaptation programmes

#### TNF-linked death checkpoints: apoptosis and necroptosis

A20 has emerged as an important determinant of how TNF signalling is translated into cell-death outcomes, particularly at the interface between receptor-proximal ubiquitin organization and downstream execution complexes. The most compelling evidence for apoptotic control places A20 at TNFR1 complex I, where it limits TNF-induced apoptosis through mechanistically separable modes of action. Under conditions in which linear ubiquitylation is intact, A20 stabilizes the M1-linked ubiquitin scaffold associated with complex I in a ZF7-dependent manner; when linear ubiquitylation is compromised, protection is maintained through a distinct ZF4/ZF7- and deubiquitinase-dependent mechanism, indicating that A20 acts not simply as a generic survival factor but as a context-sensitive organizer of ubiquitin-dependent signalling at the receptor complex [76]. Yet this function is not unidirectional. In intestinal epithelial cells, increased A20 expression can instead sensitize cells to TNF-driven, RIPK1-dependent apoptosis through ripoptosome-associated signalling in a ZnF7-dependent manner [86]. A comparable shift has been described in keratinocytes, where A20 promotes TNF-induced apoptosis by altering cIAP/NIK-dependent NF-κB signalling and facilitating ripoptosome assembly [87]. The necroptotic branch is supported most directly at the level of RIPK3: A20 restricts RIPK3 ubiquitination and limits RIPK1–RIPK3 complex formation, thereby constraining RIPK3-dependent necroptosis [88]. More recent in vivo data extend this framework by showing that A20 suppresses K63-linked ubiquitination of RIP3 in ischemic brain tissue, attenuating neuronal necroptosis and the associated pro-inflammatory polarization of microglia/macrophages [11]. This view is further reinforced by systems-level analyses indicating that inducible A20 operates within a TNF–NF-κB/RelA–RIPK3 regulatory circuit that shapes necroptotic fate in a stimulus-duration-dependent manner, suggesting that A20 contributes not only to the integrity of death checkpoints themselves but also to the temporal logic by which necroptotic decisions are made [89]. Rather than functioning as a uniformly cytoprotective brake, A20 therefore appears to tune the threshold, timing and routing of TNF-associated death signalling across multiple ubiquitin-sensitive checkpoints.

#### Inflammasome-linked inflammatory death: pyroptosis-associated outputs

A20 is now recognized as an important brake on inflammasome-linked inflammatory injury, with the most convincing evidence centred on NLRP3 rather than on a universal control of pyroptotic execution itself. In myeloid cells, A20 deficiency selectively amplifies NLRP3-dependent caspase-1 activation, IL-1β release and pyroptosis, and genetic interruption of the NLRP3–IL-1 axis markedly protects A20-deficient mice from inflammatory tissue damage [62]. This effect is not limited to priming, as A20 also associates with pro-IL-1β- and caspase-1-containing complexes and restricts RIPK3-dependent ubiquitination of these assemblies, thereby constraining aberrant inflammasome activation [90]. More recent studies extend this framework to a NEK7-dependent activation step: A20 binds NEK7, promotes its ubiquitin-dependent turnover, and interferes with NEK7 recruitment to the NLRP3 complex, thereby dampening downstream pyroptosis-associated outputs in vitro and in vivo [68]. Consistent with this, in oxidized self-DNA-driven acute kidney injury, A20 or A20-derived peptides suppress the NEK7–NLRP3 axis, reduce GSDMD-linked pyroptosis and alleviate tissue injury [3]. Human evidence is more limited but directionally aligned, as immune cells from patients with haploinsufficiency of A20 display enhanced NLRP3 responsiveness and excessive IL-1β/IL-18 secretion [91]. Thus, current data place A20 primarily as a regulator of NLRP3 activation, inflammasome assembly and IL-1 processing, whereas direct control of pyroptotic execution appears more context dependent.

#### Autophagy and survival bias as adaptive alternatives

Rather than being confined to terminal inflammatory death pathways, A20 also shapes whether stressed cells engage adaptive autophagy programmes that preserve homeostasis. In macrophages, A20 counteracts TRAF6-dependent K63-linked ubiquitination of Beclin-1 and thereby limits TLR4-induced autophagy, establishing a ubiquitin-sensitive checkpoint through which inflammatory signals are coupled to autophagosome initiation [92]. More recent work extends this principle upstream to ATG9A, showing that A20 antagonizes TRAF6-mediated K48/K63-linked ubiquitination of ATG9A and attenuates ATG9A–Beclin 1 interaction and VPS34-complex assembly during oxidative stress-induced autophagy [93]. Yet the functional consequence of A20-dependent autophagy regulation is not simply inhibitory. In activated CD4 T cells, TNFAIP3 instead promotes autophagy by restricting MTOR activity; loss of Tnfaip3 reduces LC3 puncta, increases mitochondrial content and ROS, and compromises T-cell survival, whereas MTOR inhibition rescues this defect in an ATG5-dependent manner. This survival-oriented programme is echoed in inflammatory and degenerative tissue settings, where TNFAIP3 overexpression suppresses mTOR signalling, enhances autophagy and limits extracellular-matrix loss in nucleus pulposus cells, and where more recent studies further connect A20-mediated protection to BNIP3-dependent mitophagy and stabilization of mitochondrial homeostasis [85, 94]. At the same time, these effects are not unidirectional across contexts: in hypoxic periodontal ligament cells, A20 suppresses TRAF6-dependent autophagy together with osteoclastogenic output, indicating that the relationship between A20 and autophagy is shaped by cell type and stress context rather than by a uniformly pro-autophagic function [95]. A20 therefore seems to bias stressed cells towards adaptive recovery by tuning ubiquitin-sensitive autophagy checkpoints, although the balance between autophagy, mitophagy and tissue protection remains context dependent [92].

### Oxidative stress and immunometabolic adaptation

#### Oxidative-stress control as a downstream functional module

Although early work first linked A20 to oxidative-stress responses, these studies mainly established conceptual rather than unified protective roles. In particular, cell-based experiments showed that A20 can be induced by reactive oxygen species yet, under severe oxidative stress, may also terminate NF-κB-dependent survival signalling and sensitize cells to necrotic death, indicating from the outset that its influence on oxidative-stress outcomes is context dependent [96]. Classical in vivo support subsequently came from ischemia–reperfusion models in liver and kidney, where increased A20 expression was associated with reduced inflammatory, apoptotic and necroptotic injury, placing A20 within the broader machinery that limits oxidative-stress-rich reperfusion damage [97, 98]. Recent work has moved this field beyond generic ROS-associated injury toward more defined oxidative damage products and stress-response circuits. In acute kidney injury, oxidized self-DNA accumulates in both mice and patients, functions as a damage signal that activates STING and, more prominently, NEK7–NLRP3-linked pyroptotic amplification, and induces A20 as a self-limiting regulator; mechanistically, A20 restrains this pathway by interfering with NEK7–NLRP3 complex formation, thereby attenuating downstream inflammatory injury rather than simply acting as a direct ROS scavenger [3]. A complementary recent line of evidence places A20 within Nrf2-associated stress-response networks. In cerebral ischemia–reperfusion injury, neuroprotection has been linked to a Nrf2/A20/eEF1A2 axis, supporting the view that A20 may participate in coordinated antioxidant and anti-inflammatory adaptation rather than in a standalone detoxification pathway [99]. Likewise, in lung injury models, A20 upregulation occurs together with NRF2/HO-1 activation, reduced oxidative-stress markers and suppression of NF-κB-driven inflammation, although here the evidence remains pathway-level and does not yet define direct molecular hierarchy between A20 and Nrf2 [100]. Taken together, current evidence most strongly supports A20 as a context-dependent limiter of oxidative-damage propagation and secondary inflammatory amplification, with recent advances shifting attention from bulk ROS readouts toward oxidized danger signals and integrated stress-response networks.

#### Immunometabolic rewiring, mitochondrial stress and adaptive recovery

Beyond its role in suppressing inflammatory signalling, A20 is increasingly implicated in the adaptive programmes that couple immune-cell state transitions to metabolic and mitochondrial fitness. The clearest evidence for a direct immunometabolic role comes from myeloid cells, where A20 deficiency rewires macrophage fuel handling in vivo: A20-deficient macrophages display impaired mitochondrial respiratory function, metabolize more palmitate in vitro and in vivo, and are associated with protection from diet-induced obesity and insulin resistance despite a pro-inflammatory tissue environment, indicating that A20 can shape macrophage immunometabolism at the level of fatty-acid utilization rather than simply altering cytokine output [61]. A complementary mechanistic line of evidence places A20 within MTOR-linked adaptive recovery pathways. In CD4 T cells, TNFAIP3 binds the MTOR complex, restrains MTOR activity, promotes autophagy, and limits mitochondrial accumulation and ROS, thereby sustaining cell survival; importantly, rescue by MTOR inhibition is lost in the absence of ATG5, arguing that this is an autophagy-dependent survival programme rather than a generic anti-apoptotic effect [63]. This framework extends to myeloid and stromal stress settings. In human monocytes, a TNFAIP3–DEPTOR complex promotes early autophagy and restrains inflammasome output, linking A20 to an mTOR-related adaptive module that tempers immune activation [101]. In parallel, studies in stressed nucleus pulposus cells show that TNFAIP3 suppresses mTOR signalling, promotes autophagy or BNIP3-dependent mitophagy, stabilizes mitochondrial dynamics, and reduces pyroptotic and apoptotic injury, supporting the view that A20 can redirect damaged cells away from terminal collapse toward mitochondrial quality control and recovery [85]. More recent in vivo work further suggests that myeloid A20 is required for appropriate alternative macrophage polarization, as its absence drives a persistent classically activated programme that favors type-1 immunity at the expense of type-2 adaptation [6]. Taken together, current evidence supports A20 less as a universal metabolic master regulator than as a context-dependent coordinator of immunometabolic adaptation, with the strongest support for roles in fatty-acid handling, MTOR-restrained autophagy, mitochondrial stress recovery and macrophage state programming [61, 84].

## Clinical associations and disease implications

Disruption of A20 function is clinically expressed through diverse but mechanistically connected disease phenotypes. These disorders do not reflect a single inflammatory pathway, but arise from failure of one or more A20-regulated modules, including immune tolerance, receptor-proximal signal termination, regulated cell death, barrier protection, stress adaptation and tissue remodeling [102–104]. The dominant manifestation depends on the affected cellular compartment, the mode of TNFAIP3 dysregulation and the pathological output that becomes engaged. Accordingly, this section discusses A20-associated diseases across autoimmune and autoinflammatory disorders, infections, cancer, and other inflammatory, remodeling or organ-protective conditions.

### Autoimmune and autoinflammatory diseases

Autoimmune and autoinflammatory disorders represent the most direct clinical manifestations of impaired A20 function [105]. Across these diseases, the central pathogenic event is not simply increased inflammation, but loss of the regulatory threshold that normally constrains receptor-proximal immune activation and preserves tolerance [103]. The resulting phenotypes differ according to the dominant cellular compartment, the mode of TNFAIP3 dysregulation, and the downstream tissue programme that becomes engaged [104]. In some settings, this primarily manifests as chronic cytokine amplification and systemic autoinflammation; in others, it is coupled to type I interferon dominance, compartment-specific barrier failure, or persistent stromal and vascular injury [102].

#### Haploinsufficiency of A20

Haploinsufficiency of A20 (HA20) is the most direct clinical example of disease caused by partial loss of A20 function [106, 107]. In this disorder, human genetic studies have identified heterozygous TNFAIP3 variants, including truncating mutations, single-nucleotide substitutions and small insertions or deletions, that reduce effective A20 dosage and are sufficient to cause systemic inflammatory disease [65, 108]. The causal relationship is therefore unusually direct relative to most other disorders discussed in this review. Clinically, HA20 presents with overlapping features of Behçet-like disease, lupus-like autoimmunity, psoriasis and juvenile inflammatory arthritis, indicating that partial A20 insufficiency destabilizes a broad immune checkpoint rather than a single tissue-restricted pathway [44, 107, 109]. The molecular phenotype of HA20 has been defined primarily through patient immune profiling and functional studies in patient-derived cells [1, 44]. These studies consistently report increased inflammatory mediators, including IL-1β, IL-6 and tumor necrosis factor alpha (TNF-α), together with exaggerated immune-cell activation [1, 44, 110]. Several studies have linked HA20 to heightened inflammasome-related IL-1β/IL-18 output, although the strength of direct patient-level evidence remains more limited than for broader pro-inflammatory activation [111]. Other studies have linked HA20 to increased type I interferon-associated chemokines, including C-X-C motif chemokine ligand 9 (CXCL9) and C-X-C motif chemokine ligand 10 (CXCL10), and to T helper 1 (Th1) -skewed immune responses [1, 44]. In severe inflammatory settings, RIPK1-receptor-interacting serine/threonine-protein kinase 3 (RIPK3)-mixed lineage kinase domain-like protein (MLKL)-linked necroptotic pathways have been proposed as potential amplifiers of tissue injury, but direct evidence that this axis is uniformly engaged across HA20 patients remains limited [110, 112]. These downstream outputs are biologically plausible and supported by patient-derived and disease-associated data; however, they should not be presented as uniformly present or equally dominant in every individual with HA20 [113].

#### Rheumatoid arthritis

In rheumatoid arthritis (RA), the defining pathological consequence of A20 dysregulation is the coupling of persistent synovial inflammation to osteoclast-driven structural joint damage. Earlier genetic and mouse studies first placed A20 in the RA landscape, but these now serve mainly as background for a more compartment-specific model in which A20 modifies susceptibility and tissue injury across distinct effector contexts [114]. Recent human genetics still support a contribution of TNFAIP3 variation, including rs2230926 and rs5029937, yet the strength and reproducibility of these associations vary across cohorts, arguing for a real but non-uniform genetic signal rather than a universally predictive marker [115–117]. The most clinically proximal recent evidence instead centers on the myeloid compartment: RA patients show expansion of intermediate and non-classical monocytes, with altered A20 expression correlating with anti-CCP levels and radiographic burden, while METTL14-mediated m6A regulation suggests that TNFAIP3 dysregulation extends beyond germline polymorphism [118, 119]. Functional studies are broadly consistent with this interpretation, as TNFAIP3 perturbation modifies monocyte migration, IL-6/MMP output and macrophage polarization in experimental arthritis, although these data remain supportive rather than definitive for patient pathogenesis [120]. The clearest recent mechanistic advance concerns the structural phenotype itself: A20 is recruited to the RANK receptor complex and restrains RANK-induced NF-κB signaling through its ZnF4/ZnF7 ubiquitin-binding functions, providing a direct basis for limiting osteoclastogenesis and bone resorption [33]. By contrast, links to synoviocyte-associated inflammasome or pyroptosis-related outputs remain largely pathway-level and are less securely established as dominant A20-dependent drivers in RA [121].

#### Behçet’s disease

Behçet’s disease is a relapsing multisystem inflammatory vasculitic syndrome characterized by recurrent oral and genital ulceration, ocular inflammation, skin lesions, and variably articular or visceral involvement [122]. Early Chinese studies implicated TNFAIP3 polymorphisms in disease susceptibility, yet this association was not replicated in a European cohort, indicating that common-variant evidence for conventional BD is ancestry-dependent and appreciably less secure than is sometimes implied [123]. By contrast, TNFAIP3 loss-of-function defines A20 haploinsufficiency (HA20), an early-onset Behçet-like autoinflammatory disease in which reduced A20 expression is coupled to enhanced NF-κB signaling and increased inflammatory cytokine output [124]. Recent sequencing studies strengthen the clinical relevance of this framework by showing that a substantial minority of patients labelled as suspected Behçet’s disease instead harbor monogenic mimics, with HA20 emerging as a prominent diagnosis; importantly, rare TNFAIP3 missense variants are not uniformly pathogenic and may require functional validation before being treated as disease-defining evidence [36, 122]. Overall, the strongest case for A20 in the Behçet spectrum lies in monogenic mucosal-autoinflammatory disease, whereas its contribution to classical polygenic Behçet’s disease remains more limited and context dependent.

#### Systemic lupus erythematosus

Systemic lupus erythematosus (SLE) is a multisystem autoimmune disease defined by immune-complex injury, a dominant type I interferon milieu and organ-selective manifestations such as nephritis and neuropsychiatric disease [125, 126]. Earlier genetic work first placed TNFAIP3 within the lupus susceptibility landscape, whereas more recent studies show that this signal is mechanistically layered at both chromatin and RNA levels. Specifically, the SLE risk haplotype at the TNFAIP3 locus can remodel long-range enhancer activity to suppress TNFAIP3 while increasing IFNGR1 and IL20RA, embedding A20 deficiency within a broader IFN-responsive regulatory network rather than an isolated NF-κB defect [127]. A further post-transcriptional layer is added by circGARS, which promotes SLE-associated immune activation through a miR-19a–YTHDF2–A20/NF-κB axis in patient PBMCs [51]. The disease relevance of A20 becomes most compelling in high-effect phenotypes, as systematic synthesis of HA20 cases shows that a small but informative subset of apparent SLE represents TNFAIP3-related monogenic or near-monogenic disease, often enriched for early onset, fever, mucosal ulcers, renal involvement and neuropsychiatric features [125]. More broadly, recent review-level evidence places tissue-resident memory T cells among compartmental amplifiers of type I IFN responses and autoantibody production in SLE, providing a useful tissue-context framework for interpreting how A20 insufficiency may be expressed within persistent lesional immune niches [128].

#### Multiple sclerosis

Multiple sclerosis (MS) is a chronic inflammatory demyelinating disease of the central nervous system in which barrier dysfunction, immune-cell trafficking and tissue injury are tightly coupled [129]. Human neuropathology shows that A20 is increased within active MS lesions, including active and chronic active white-matter lesions and active cortical lesions, where it is enriched in perivascular macrophages, activated astrocytes and microglia; notably, this central upregulation contrasts with previously reported peripheral downregulation, arguing that CNS and systemic compartments do not simply mirror the same A20 biology [130, 131]. The strongest mechanistic evidence comes from recent CNS endothelial A20-deficiency models, in which loss of A20 enhances EAE and increases endothelial ICOSL, ICAM-1 and VCAM-1, thereby promoting lymphocyte adhesion and entry into the CNS; importantly, this points less to a generic anti-cytokine role than to failure of barrier-associated immune restraint at the vascular gate [105].

#### Psoriasis

Psoriasis is a chronic inflammatory skin disease in which epidermal hyperplasia, altered keratinocyte differentiation and immune-cell recruitment are tightly coupled [103]. In psoriasis-associated CARD14^E138A signalling, A20 and ABIN1 are recruited as negative regulators that promote CARD14 turnover and thereby restrain NF-κB and MAP kinase activation, whereas mTORC1 constitutes a partially separable downstream output linked more closely to keratinocyte metabolism, proliferation and acanthosis; concordantly, rapamycin attenuates CARD14-driven epidermal thickening without abolishing inflammatory recruitment, arguing that inflammatory transcriptional programmes and hyperproliferative remodelling can be at least partly uncoupled within the epidermal compartment [39]. This epithelial restraint model is reinforced by a recent keratinocyte-specific A20-deficiency study, in which loss of A20 unleashes MyD88-dependent signalling and is sufficient to initiate psoriasiform skin inflammation, whereas subsequent disease propagation requires T cells, indicating that failure of epidermal A20 control can convert keratinocytes from passive targets into initiating inflammatory effectors [104]. By contrast, patient-linked evidence is more associative but directionally consistent: lesional skin and TNFα-stimulated keratinocytes show reduced UCA1, heightened NF-κB activity and lower A20 expression, consistent with a UCA1–miR125a–A20 regulatory axis that may weaken epithelial restraint of inflammatory signalling, although this route is better viewed as a modulatory mechanism than as a fully resolved causal pathway [49]. At the systemic level, peripheral-blood studies further associate reduced A20 expression with psoriasis and with selected immune–metabolic correlates, but these findings derive from the circulating compartment and should not be overextended into direct claims about lesional epithelial mechanism [81].

### Infectious diseases

In infection, the biological role of A20 is shaped by a central tension between host protection and pathogen exploitation [6, 102]. On the one hand, A20 limits excessive inflammatory signalling, tissue injury and inflammatory cell death, thereby protecting the host from immunopathology [132]. On the other hand, the same restraint can be co-opted by pathogens to weaken antimicrobial responses, prolong intracellular survival or preserve a permissive tissue niche [133–135]. Therefore, A20 cannot be interpreted as uniformly protective or uniformly pathogenic. Its net effect depends on the cellular compartment involved, the pathogen’s mode of host interaction, and whether the dominant selective pressure is inflammatory tissue damage or pathogen clearance [6, 102, 134]. Representative non-neoplastic disease contexts associated with A20 dysregulation, including their underlying mechanisms and pathological consequences, are summarized in Table 2.

**Table 2:** Representative non-neoplastic disease associations of A20

Disease category	Representative disease/context	Mechanism of A20 dysregulation/action	Clinical/pathological implications	Reference
Autoimmune/autoinflammatory diseases	Haploinsufficiency of A20 (HA20)	Heterozygous TNFAIP3 variants reduce effective A20 dosage, lower the inflammatory threshold, and amplify NF-κB-, inflammasome-, type I IFN- and RIPK1–RIPK3–MLKL-associated outputs	Systemic autoinflammation with Behçet-like, lupus-like, psoriatic and juvenile inflammatory arthritis-like manifestations	[1, 44, 65, 108, 110–112]
Autoimmune/autoinflammatory diseases	Rheumatoid arthritis	TNFAIP3 variation and altered A20 expression affect myeloid activation, IL-6/MMP output and macrophage polarization; A20 restrains RANK-induced NF-κB signaling through ZnF4/ZnF7-dependent ubiquitin-binding functions	Persistent synovial inflammation coupled to osteoclastogenesis, bone resorption and structural joint damage	[114–121]
Autoimmune/autoinflammatory diseases	Behçet’s disease/Behçet-like disease	TNFAIP3 loss-of-function defines HA20-associated Behçet-like autoinflammation, whereas common-variant evidence for classical Behçet’s disease is ancestry-dependent and less secure	Early-onset mucosal autoinflammation with recurrent ulceration and systemic inflammatory features; limited and context-dependent contribution to classical polygenic Behçet’s disease	[122–124]
Autoimmune/autoinflammatory diseases	Systemic lupus erythematosus	TNFAIP3 risk haplotypes and RNA-level regulation suppress A20 within broader IFN-responsive regulatory networks, including enhancer remodeling and circGARS–miR-19a–YTHDF2–A20/NF-κB signaling	IFN-dominant autoimmunity, immune-complex injury, nephritis/neuropsychiatric involvement and selected HA20-associated SLE-like disease	[125–128]
Autoimmune/autoinflammatory diseases	Multiple sclerosis	CNS endothelial A20 deficiency enhances EAE and increases ICOSL, ICAM-1 and VCAM-1, promoting leukocyte adhesion and entry into the CNS	Neurovascular barrier dysfunction, immune-cell trafficking and compartment-specific neuroinflammation	[129–131]
Autoimmune/autoinflammatory diseases	Psoriasis	Keratinocyte A20 restrains CARD14-, MyD88-, NF-κB- and MAPK-associated epithelial inflammatory signaling; reduced UCA1–miR125a–A20 regulation may weaken lesional epithelial restraint	Epidermal hyperplasia, psoriasiform inflammation, altered keratinocyte differentiation and immune-cell recruitment	[39, 41, 81, 103, 104]
Infectious diseases	Viral infections	In HBV/HCV, A20 dampens myeloid antiviral activation; in HRSV, HCoV-229E and influenza A virus, A20 restrains epithelial antiviral/inflammatory signaling and can support viral persistence or replication; ALV-A links A20 to TRAF6–STAT3–c-Myc pathogenic signaling	Pathogen immune evasion, weakened antiviral cytokine responses, epithelial survival bias, viral persistence and selected tissue-injury or oncogenic outputs	[133, 134, 136–140]
Infectious diseases	Helicobacter pylori infection	H. pylori induces epithelial A20, which terminates classical NF-κB signaling, modulates apoptosis and restrains alternative NF-κB activity through TIFA–NIK/RelB-related signaling	Calibration of gastric epithelial inflammation and survival, potentially limiting tissue damage while maintaining a niche permissive for persistent infection	[141–144]
Infectious diseases	Mycobacterium tuberculosis infection	Mtb-associated circuits derepress or regulate A20 in macrophages, attenuating NF-κB-linked TNF, IL-1β and nitric oxide production and influencing apoptosis–necrosis balance through RIPK3-related mechanisms	Altered macrophage antimicrobial activity, enhanced intracellular bacillary survival, modified cell-death routing and possible dissemination-associated inflammatory states	[135, 145–149]
Infectious diseases	Parasitic infections	In Giardia infection, A20 promotes NLRP3 deubiquitination and inflammasome-associated pyroptosis; in Leishmania infantum, A20 dampens NF-κB and NLRP3 priming in microglia	Species- and compartment-dependent effects, ranging from host-defence-associated inflammasome activation to immune-subversive anti-inflammatory responses	[150–154]
Infectious diseases	Fungal infections	In systemic candidiasis, A20 restrains CLR-dependent NF-κB/JNK cytokine signaling; autophagy can transiently lift A20-dependent suppression, whereas in Aspergillus keratitis A20 appears more tissue-protective	Context-dependent control of antifungal defense, neutrophil recruitment, macrophage inflammatory output and local immunopathology	[155–157]
Infectious diseases/microbial regulation	Microbial metabolites and probiotics	Itaconic acid, microbial metabolites and selected probiotics modulate A20-associated NF-κB or immunometabolic regulatory signatures, although A20 is not always the decisive causal node	Tuning of macrophage or epithelial inflammatory responses, mucosal injury, oxidative stress and antibacterial/antiviral immune balance	[158–165]
Barrier and epithelial inflammatory disorders	Inflammatory bowel disease	A20 preserves intestinal epithelial barrier competence by limiting TNFR1-, RIPK1/RIPK3/Caspase-8- and inflammatory permeability-associated injury; miR-23a-mediated TNFAIP3 repression and ATG16L1 interaction further modulate this axis	Chronic intestinal inflammation, epithelial vulnerability, increased permeability and barrier failure	[166–170]
Barrier and epithelial inflammatory disorders	Airway–mucosal inflammatory disorders	Reduced epithelial A20 amplifies alarmin-driven type 2 signaling, including IL-33, TSLP and IL-25, and contributes to epithelial–mesenchymal transition and mucosal remodeling	Asthma, allergic rhinitis and chronic rhinosinusitis with persistent airway inflammation, barrier dysfunction and remodeling	[171–175]
Barrier and epithelial inflammatory disorders	Periodontal inflammatory disease	Reduced gingival or periodontal A20 weakens negative feedback on inflammatory signaling; A20 promotes autophagy, reduces pyroptosis and modulates TRAF6/RANKL–OPG-associated osteoclastogenic remodeling	Gingival inflammation, epithelial injury, pyroptotic inflammatory output and alveolar bone destruction	[176–181]
Barrier and epithelial inflammatory disorders	Skin inflammatory disorders	A20 restrains keratinocyte inflammatory activation, adhesion molecule expression, epithelial alarmins and NLRP3-associated stress responses; zinc/formononetin and GPER1-related pathways may modulate A20 activity	Atopic dermatitis-like inflammation, acne-associated keratinocyte inflammation and AGE–RAGE-associated skin stress or pigmentation-related phenotypes	[182–185]
Fibrotic and inflammatory remodeling	Pulmonary fibrosis	Reduced A20 activity facilitates TGF-β/Smad2/3 signaling, myofibroblast differentiation, collagen deposition, ROS accumulation and NLRP3/IL-33-associated amplification	Fibroproliferative remodeling, epithelial injury and progression from chronic inflammation to stromal fibrogenesis	[186–188]
Fibrotic and inflammatory remodeling	Renal inflammatory and fibrotic disease	A20 is linked to the PGC-1α–TNFAIP3 axis, mitochondrial fitness and NLRP3 restraint; miR-29b and exosomal miR-21-5p can suppress TNFAIP3 in diabetic or inflammatory renal contexts	Renal fibrosis, podocyte injury, tubular stress, oxidative injury and inflammasome-associated renal inflammation	[189–193]
Fibrotic and inflammatory remodeling	Hepatic inflammatory and fibrotic disease	A20 abundance is regulated by protein stability, including RNF31-mediated ubiquitination and degradation; A20 also intersects with NOD1-associated innate signaling and metabolic liver disease circuits	Acute hepatic inflammatory/oxidative injury, chronic liver remodeling, fibrosis and context-dependent metabolic liver disease progression	[194–199]
Bone–cartilage inflammatory remodeling	Osteoarthritis	TNFAIP3 from osteoarthritic cartilage or skeletal stem-cell contexts modulates osteoclast formation, subchondral bone remodeling and osteoblast necroptosis; miR93/TLR/NF-κB and AMPK/SIRT1 pathways provide supportive pathway-level evidence	Abnormal osteochondral remodeling, subchondral bone pathology and structural joint degeneration	[167, 200, 201]
Bone–cartilage inflammatory remodeling	Intervertebral disc degeneration	A20 restrains mTOR signaling, promotes BNIP3-associated mitophagy/autophagy and preserves extracellular matrix integrity in nucleus pulposus cells	Matrix loss, impaired disc homeostasis and inflammatory or degenerative disc injury when A20 is reduced	[63, 94, 202]
Bone–cartilage inflammatory remodeling	Osteoporosis	Osteoclast-specific TNFAIP3 deletion causes severe osteoporosis; A20 restrains RANK-induced osteoclast differentiation mainly through ZnF4/ZnF7-mediated ubiquitin-binding functions	Excessive osteoclastogenesis, bone resorption and osteoporosis independent of overt systemic inflammatory arthritis	[33, 203, 204]
Bone–cartilage inflammatory remodeling	Ankylosing spondylitis	Reduced TNFAIP3 enhances Wnt/β-catenin signaling and osteogenic differentiation; MEG3/miR-125a-5p/TNFAIP3 regulation may limit aberrant ossification	Inflammation-associated pathological new bone formation and ankylosing-spondylitis-related ossification	[205, 206]
Vascular and cardiac inflammatory remodeling	Atherosclerosis	A20 preserves endothelial homeostasis through ERK5–KLF2–eNOS signaling and suppresses oxLDL/NF-κB-driven macrophage inflammation, foam-cell formation and adhesion molecule expression	Reduced endothelial injury, monocyte adhesion, vascular inflammation and plaque progression	[55, 207–211]
Vascular and cardiac inflammatory remodeling	Other cardiac inflammatory conditions	TNFAIP3 downregulation through miR-1a-3p or miR-218-5p is linked to cardiomyocyte inflammatory injury, apoptosis and TGF-β-associated remodeling	Viral myocarditis- or dilated cardiomyopathy-associated myocardial injury, apoptosis and fibrosis	[52, 212]
Neuroimmune and hematological contexts	Neurological disorders	A20 preserves blood–brain barrier integrity and limits inflammasome- or RIPK3-associated neuroinflammatory injury; MALT1-mediated A20 degradation can enhance ischemia–reperfusion damage	Neurovascular barrier disruption, neuroinflammation, ischemic injury and early brain injury after hemorrhage	[29, 151, 213–215]
Neuroimmune and hematological contexts	Hematological inflammatory disorders	Reduced A20 affects hematopoietic stem/progenitor cell resilience, myeloid-biased differentiation and inflammatory cytokine states; ABIN1–TNFAIP3 cooperation limits type I IFN-associated cytopenic phenotypes	Hematopoietic imbalance, inflammatory microenvironment, myelodysplastic-syndrome-associated inflammation and cytopenic phenotypes	[216–218]
Immunometabolic and endocrine disorders	Diabetes, vascular calcification and obesity	A20 protects β-cell integrity and islet graft survival, restrains high-glucose-induced vascular smooth-muscle osteogenic conversion through zinc/GPR39 signaling, and has context-dependent roles in macrophage fatty-acid metabolism during obesity	β-cell inflammatory injury, graft vulnerability, vascular calcification, insulin resistance or obesity-related metabolic adaptation depending on cellular compartment	[61, 219–221]

*A20* tumor necrosis factor alpha-induced protein 3, *TNFAIP3* tumor necrosis factor alpha-induced protein 3, *NF-κB* nuclear factor kappa B, *IFN* interferon, *RIPK1* receptor-interacting serine/threonine-protein kinase 1, *RIPK3* receptor-interacting serine/threonine-protein kinase 3, *MLKL* mixed lineage kinase domain-like protein, *IL-6* interleukin-6, *MMP* matrix metalloproteinase, *ZnF* zinc-finger, *RANK* receptor activator of nuclear factor kappa-B, *SLE* systemic lupus erythematosus, *CNS* central nervous system, *EAE* experimental autoimmune encephalomyelitis, *ICOSL* inducible T-cell co-stimulator ligand, *ICAM-1* intercellular adhesion molecule 1, *VCAM-1* vascular cell adhesion molecule 1, *CARD14* caspase recruitment domain-containing protein 14, *MAPK* mitogen-activated protein kinase, *MyD88* myeloid differentiation primary response 88, *HBV* hepatitis B virus, *HCV* hepatitis C virus, *HRSV* human respiratory syncytial virus, *HCoV-229E* human coronavirus 229E, *ALV-A* avian leukosis virus subgroup A, *TRAF6* TNF receptor-associated factor 6, *STAT3* signal transducer and activator of transcription 3, *TIFA* TRAF-interacting protein with forkhead-associated domain, *NIK* NF-κB-inducing kinase, *RelB* RelB proto-oncogene, *NF-κB* subunit, *Mtb* Mycobacterium tuberculosis, *NLRP3* NOD-like receptor family pyrin domain-containing 3, *CLR* C-type lectin receptor, *JNK* c-Jun N-terminal kinase, *TNFR1* tumor necrosis factor receptor 1, *Caspase-8* cysteine-aspartic acid protease 8, *ATG16L1* autophagy-related 16 like 1, *TSLP* thymic stromal lymphopoietin, *IL-25* interleukin-25, *TGF-β* transforming growth factor beta, *Smad2/3* SMAD family member 2/3, *ROS* reactive oxygen species, *PGC-1α* peroxisome proliferator-activated receptor gamma coactivator 1-alpha, *RNF31* ring finger protein 31, *NOD1* nucleotide-binding oligomerization domain-containing protein 1, *BNIP3* BCL2 interacting protein 3, *Wnt/β-catenin* Wnt/beta-catenin signaling pathway, *MEG3* maternally expressed 3, *miR-125a-5p* microRNA-125a-5p, *ERK5* extracellular signal-regulated kinase 5, *KLF2* Kruppel-like factor 2, *eNOS* endothelial nitric oxide synthase, *oxLDL* oxidized low-density lipoprotein, *GPER1* G protein-coupled estrogen receptor 1, *AGE* advanced glycation end product, *RAGE* receptor for advanced glycation end products, *AMPK* AMP-activated protein kinase, *SIRT1* sirtuin 1, *MALT1* mucosa-associated lymphoid tissue lymphoma translocation protein 1, *ABIN1* A20-binding inhibitor of NF-κB 1, *GPR39* G protein-coupled receptor 39

#### Viral infections

Viral infection illustrates particularly clearly that A20 cannot be assigned a fixed protective or pathogenic role. Instead, its significance is best understood in relation to the dominant pathological output and the cellular compartment in which the virus engages the host [9]. Across current studies, the most consistent theme is that A20 acts as a brake on innate antiviral signalling that can be co-opted to support viral persistence, although the consequences of this restraint differ between myeloid cells and infected epithelia [134, 136–138].

This pattern is most clearly resolved in the myeloid compartment, where A20 is linked to attenuated antiviral activation rather than to direct control of viral replication [134, 136]. In HBV, HBsAg induces A20 in monocytes, suppresses TRAF6 polyubiquitination and impairs TRAF6–TA B2 complex formation, thereby weakening TLR4 responsiveness and pro-inflammatory cytokine production; A20 silencing partially restores these responses, supporting a role in monocyte immune evasion [134]. A related, although less direct, configuration is seen in HCV, where viral antigen exposure upregulates A20 and ABIN1, suppresses NF-κB activity and compromises M1-like macrophage polarization, consistent with a shift away from inflammatory antiviral programming [136].

Moving from myeloid cells to the infected epithelial compartment, A20 again tends to support a permissive state, but here the output is shaped by the balance between antiviral signalling, cell survival and productive replication [133, 137, 138]. In HRSV-infected airway epithelial cells, A20 and its associated factors TAX1BP1 and ABIN1 dampen early antiviral and inflammatory responses, reduce apoptosis and favour viral production [133]. HCoV-229E shows a related but more transcriptionally complex phenotype: A20 is induced while inducible NF-κB activity is simultaneously constrained through partial loss of IKKβ, NEMO and IκBα, suggesting that coronavirus-infected cells maintain a calibrated level of signalling that supports replication while avoiding excessive inflammatory activation [137]. Influenza A virus extends this principle further, as NS1-induced A20 suppresses IRF3-driven interferon responses and thereby facilitates viral replication in infected epithelial cells [138].

Beyond suppression of innate immune signalling, some viral settings indicate that A20 can also feed into broader pathogenic programmes [139]. In ALV-A infection, A20 not only aggravates viraemia, shedding and tissue injury in vivo, but also promotes a TRAF6–STAT3–c-Myc axis in infected DF-1 cells by reducing TRAF6 ubiquitination, linking A20 to oncogenic signalling as well as to viral pathogenicity [140]. Viewed together, these studies suggest that A20 in viral disease is most usefully interpreted not as a uniform antiviral effector or a simple proviral factor, but as a context-sensitive regulator whose net effect emerges from the interaction between viral strategy, cell type and pathological output [9].

#### Bacterial infections

##### Helicobacter pylori

In *Helicobacter pylori* infection, A20 is most usefully understood as a regulator of the gastric epithelial response that links inflammatory resolution to cell-fate control, rather than as a purely anti-inflammatory factor [142, 144]. Early work showed that *H. pylori* induces NF-κB-dependent A20 expression in gastric epithelial cells, and that A20 both terminates classical NF-κB signalling and suppresses caspase-8-dependent apoptosis, thereby favouring epithelial cell survival [142]. More recent studies have refined this model by showing that A20 also restrains alternative NF-κB activity through interference with the TIFA–NIK axis, limiting RelB-dependent anti-apoptotic gene expression, while an upstream USP48–RelA pathway prolongs A20 induction and reinforces this survival programme [141, 143, 144]. Consistent with these experimental findings, A20 expression is increased in human HP-gastritis biopsies, supporting its relevance in active gastric disease [56]. Taken together, A20 primarily acts within the gastric epithelial niche to calibrate the balance between inflammation and survival, a function that may help limit overt tissue damage but also preserve the cellular context permissive for persistent infection [56, 141, 142, 144].

##### Mycobacterium tuberculosis

Tuberculosis places A20 at a particularly unstable boundary between host defence and pathogen accommodation. In macrophages, Mtb suppresses let-7f in an ESAT-6-dependent manner, thereby derepressing A20, attenuating NF-κB activity and reducing TNF, IL-1β and nitric oxide production, with enhanced intracellular bacillary survival [147]. A20 also recurs in other macrophage regulatory circuits, including the XIST–miR-125b-5p–A20–NF-κB axis, in which reduced A20 restraint is associated with stronger NF-κB activation, enhanced M1 polarization and improved antimycobacterial activity [135]. At the level of cell fate, Fu, et al. further placed A20 within the miR-342-3p/SOCS6 pathway that influences the switch between Mtb-induced apoptosis and necrosis through RIPK3-directed regulation, indicating that A20 can also participate in death-mode control rather than simply suppress inflammation [146]. Beyond these better-supported macrophage mechanisms, newer studies have linked *TNFAIP3* to dissemination-associated inflammatory programmes in extrapulmonary tuberculosis [145], identified *TNFAIP3* as a candidate ferroptosis-related TB gene [148], and, in a Mycobacterium smegmatis model, associated A20 upregulation with ROS-dependent intracellular killing [149]; however, these latter findings remain more suggestive than definitive for Mtb itself. Currently, A20 in tuberculosis acts mainly as a pathway-specific regulator within the macrophage niche, most clearly damping NF-κB-linked antimicrobial programmes, but also intersecting with cell-death control and broader disease-associated transcriptional states.

#### Parasitic infection

Parasitic infection highlights the context dependence of A20 particularly well, as its role diverges between parasite species and inflammatory compartments. In Giardia infection, Giardia duodenalis and its secreted PPIB activate TLR4-dependent ROS signalling, induce A20, and promote A20-mediated NLRP3 deubiquitination, thereby driving caspase-1 activation, GSDMD cleavage and IL-1β/IL-18 release during macrophage pyroptosis [153]. This A20-linked inflammasome response sits within a broader Giardia-induced ROS–MAPK/NF-κB–pro-inflammatory/NO axis that is consistent with host defence, although also compatible with local inflammatory injury [154]. At the same time, the functional meaning of this pathway is sharpened by complementary studies showing that NLRP3 activation decreases Giardia pathogenicity in mice, whereas the parasite-derived VSPAS7 protein suppresses NLRP3-dependent pyroptosis and inflammatory signalling, indicating that Giardia actively counteracts this host response [150, 152]. In contrast, in Leishmania infantum, A20 is linked to the opposite output: in Aβ-activated microglia, parasite infection upregulates A20, dampens NF-κB signalling, inhibits NLRP3 priming, reduces ASC speck formation, caspase-1 activation, ROS, IL-1β and IL-18, and thereby supports an immune-subversive anti-inflammatory state [151].

#### Fungal infection

In systemic candidiasis, A20 mainly emerges as a restraint on antifungal innate signaling: Liang and colleagues showed that it terminates acute C-type lectin receptor (CLR) responses downstream of Dectin-1, Dectin-2 and Mincle, limits NF-κB/JNK-associated cytokine production, and, when reduced even by a single allele, permits stronger host defense and prolonged survival after systemic Candida albicans infection [155]. A mechanistic foundation for this view had already come from the finding that autophagy can transiently lift A20-dependent suppression in F4/80^hi tissue macrophages by sequestering A20 through p62, thereby enhancing NF-κB activity, chemokine release and neutrophil recruitment during Candida challenge [157]. A different picture is seen in Aspergillus fumigatus keratitis, where A20 was reported to lessen corneal inflammation by promoting autophagy while suppressing NF-κB signaling, placing it closer to a tissue-protective regulator of local immunopathology [21]. The inflammatory pathways involved are themselves not unidirectional in consequence, as GSDMD-dependent pyroptotic responses in candidiasis can be hijacked by C. albicans to facilitate escape from macrophages, even though inflammasome-dependent IL-1β remains beneficial to antifungal defense [156].

#### Microbial metabolites and probiotics

In macrophages, itaconic acid promotes the pentose phosphate pathway, increases ROS, induces A20, and lowers IL-6, IL-1β and TNF-α, placing A20 within an immunometabolic anti-inflammatory programme that is coupled to antibacterial activity [163]. Recent work has broadened this picture without always placing A20 at the center of the mechanism. Probiotic-derived small molecules such as tryptophol acetate and tyrosol acetate inhibit hyperinflammation in association with increased A20 expression and reduced NF-κB signaling [158], while dietary fiber-derived microbial butyrate has also been linked, at the transcriptomic level, to increased A20 within inhibitory ILC2 programmes [165]. By contrast, probiotic studies more often place A20 within a broader regulatory signature than establish it as the unique causal node. In Caco-2 cells, prophylactic Limosilactobacillus reuteri E is associated with a higher A20/IL-10 versus IL-1β/IL-8 profile during 5-FU-induced mucositis [160], whereas Lactobacillus delbrueckii-derived exopolysaccharides enhance epithelial antiviral responses in parallel with reduced A20 expression, indicating that beneficial microbial products do not invariably act by increasing A20 [164]. A similar caution applies to more recent probiotic work in epithelial and myeloid models: Lactobacillus reuteri SBC5-3 suppresses TNF-α-induced epithelial inflammation while downregulating a broader NF-κB/*TNFAIP3* transcriptional module [161]; probiotic secretomes can attenuate PCB-induced epithelial inflammation and oxidative stress, although A20 has not yet been established as the decisive mediator in that setting [159]; and a very recent THP-1 study suggests that Lacticaseibacillus paracasei KBL382 raises A20, SOCS1 and, under LPS stimulation, IRAK3, consistent with coordinated tuning of both activating and braking arms of innate signaling [162].

### Cancer

A20 exerts highly context-dependent effects in cancer [222–224]. In some tumour settings, reduced A20 expression or function is associated with persistent inflammatory signalling, enhanced invasion, metabolic reprogramming and tumour progression, consistent with a tumour-suppressive role [13, 224]. In other settings, increased or rewired A20 activity supports stress tolerance, immune evasion, or resistance to treatment, indicating that A20 can also facilitate tumour persistence under selective pressure [223, 225–227]. The critical issue is therefore not whether A20 is uniformly tumor suppressive or uniformly tumor promoting, but rather the contextual delineation of its tumor cell-intrinsic activities, microenvironmental modulation, and therapy-induced stress adaptation [228]. Representative cancer-associated roles of A20 across different tumour types are summarized in Table 3.

**Table 3:** Representative cancer-associated roles of A20

Tumor Type	A20 Pattern	Mechanism	Clinical/pathological implications	Reference
Hepatocellular carcinoma	Predominantly tumour-suppressive	A20 restricts metastatic competence through suppression of mTORC2/Akt/Rac1 signaling and limits glycolytic adaptation through PFKL-associated regulation; TNFAIP3/HSP90-associated modulation further links A20 to sorafenib response	Reduced A20 expression is associated with more aggressive, metabolically reprogrammed disease, whereas preserved A20 activity limits invasion, metastasis and glycolytic adaptation	[8, 229, 230]
Melanoma	Tumour-promoting when elevated	Elevated A20 supports Akt-dependent survival, promotes invasive behaviour, increases PD-L1 expression and weakens antitumour T-cell activity	Enhanced tumour-cell survival, immune escape, vemurafenib resistance and reduced efficacy of anti-PD-1 therapy	[224, 228]
Non-small-cell lung cancer	Context-dependent	A20 modulates TBK1–STAT1-dependent IFN-γ sensitivity, PD-L1 induction and CD8⁺ T-cell surveillance; DDR-related effects also influence cisplatin responsiveness	Coexistence of tumour-suppressive and immune-regulatory A20 functions, with effects on aggressive clinical behaviour and treatment response	[13, 225]
OSCC/NPC	Predominantly linked to genome-stress and therapy-response regulation	A20 regulates RNF168-dependent histone ubiquitination and DNA-damage response pathways, thereby influencing sensitivity to radiotherapy and platinum-based chemotherapy	Altered response to genotoxic therapy; these cancers anchor the DDR-associated tumour-intrinsic branch of A20 biology	[231–233]
Prostate cancer	A20-associated microenvironmental remodeling	Altered A20 signaling regulates the IKKβ/ARID1A/NF-κB axis and CXCR2 ligand expression, promoting recruitment of suppressive myeloid cells	Formation of an immune-evasive myeloid niche and tumour microenvironment remodeling	[234]

*A20* tumor necrosis factor alpha-induced protein 3, *TNFAIP3* tumor necrosis factor alpha-induced protein 3, *mTORC2* mechanistic target of rapamycin complex 2, *Akt* protein kinase B, *Rac1* Ras-related C3 botulinum toxin substrate 1, *PFKL* phosphofructokinase liver type, *HSP90* heat shock protein 90, *PD-L1* programmed death-ligand 1, *PD-1* programmed cell death protein 1, *TBK1* TANK-binding kinase 1, *STAT1* signal transducer and activator of transcription 1, *IFN-γ* interferon gamma, *CD8* cluster of differentiation 8, *DDR* DNA damage response, *OSCC* oral squamous cell carcinoma, *NPC* nasopharyngeal carcinoma, *RNF168* ring finger protein 168, *IKKβ* IκB kinase beta, *ARID1A* AT-rich interaction domain-containing protein 1 A, *NF-κB* nuclear factor kappa B, *CXCR2* C-X-C chemokine receptor type 2

#### The tumor-suppressive and tumor-promoting roles of A20

A20 in cancer is best interpreted through lineage, compartment and dominant stress context rather than through a fixed tumor-suppressive or tumor-promoting label [13, 223, 225]. In inflammation-associated tumorigenesis, A20 can limit the transition from chronic tissue injury to malignant transformation, as illustrated in colitis-associated cancer, where it restrains pathogenic inflammatory signaling, and in hepatocellular carcinoma, where reduced A20 is linked to glycolytic rewiring and tumour progression [224, 235]. Yet once tumours are established, A20 may be repurposed into programmes that favour persistence under selective pressure: in lung adenocarcinoma, loss of tumour-cell A20 promotes immune escape, whereas in colorectal cancer and melanoma, elevated or rewired A20 activity has been associated with impaired antitumour immunity, weaker CD8⁺ T-cell function and reduced responsiveness to PD-1 blockade [13, 223, 225]. A20 also intersects with treatment adaptation more directly, as separate work implicates it in the DNA damage response and resistance to DNA-damaging therapy [226]. Recent cross-compartment analyses further suggest that the biological consequence of targeting A20 depends on whether the dominant effect is exerted in malignant cells or in T cells. Thus, the clinical significance of A20 in cancer lies less in a single invariant role than in the signalling architecture of the tumour and stromal compartments in which it operates [235].

#### Tumour-intrinsic A20

One of the clearest tumour-intrinsic dimensions of A20 biology is its role in handling genome stress [226, 232]. Broad mechanistic work has identified A20 as a regulator of the DNA-damage response and of resistance to DNA-damaging therapy, and this function is recapitulated in nasopharyngeal carcinoma, where altered A20 expression influences radiosensitivity in tumour-cell perturbation models [226, 232]. By contrast, support for a fully resolved A20-centred DDR module in oral squamous cell carcinoma remains more limited and is currently stronger at the level of clinical association than of direct repair-pathway dissection [233]. A second, better-structured axis is seen in hepatocellular carcinoma, in which A20 suppresses both glycolytic rewiring through PFKL and invasion-associated signalling through the mTORC2/Akt/Rac1 pathway, supporting a tumour-suppressive role in metabolic and metastatic competence [224, 230]. A third tumour-intrinsic function emerges under therapy-associated stress: in acute myeloid leukaemia, elevated A20 is linked to anthracycline resistance, whereas loss of A20 restores drug sensitivity by permitting necroptotic cell death [236]. Taken together, current evidence most convincingly places tumour-intrinsic A20 at the intersection of genome-stress handling, metabolic and invasive signalling, and resistance to cell-death-inducing therapy; however, these functions are cancer-type specific and should not be generalized beyond the tumour systems in which they have been directly tested [224, 226, 230, 236].

#### A20 in the tumour microenvironment

A20 shapes tumour progression not only within malignant cells but also through the immune microenvironment, where its most recurrent outputs are immune evasion, PD-L1-associated signalling and attenuation of cytotoxic immune surveillance [13, 223, 225]. In melanoma, elevated A20 is associated with reduced CD8⁺ T-cell antitumour activity and poorer response to anti-PD-1 therapy, at least in part through prohibitin degradation, STAT3 activation and PD-L1 upregulation, while parallel work suggests that A20 can also reinforce tumour persistence through Akt-linked survival programmes [225, 228]. In lung adenocarcinoma, however, the directionality is less straightforward, as tumour-cell loss of A20 promotes tumourigenesis yet also drives immune escape through dysregulated TBK1–STAT1 signalling, increased IFN-γ sensitivity and PD-L1 induction, with consequent reduction in CD8⁺ T-cell surveillance. A distinct microenvironmental mechanism is seen in prostate cancer, where reduced A20 signalling downstream of ARID1A loss enhances NF-κB-dependent CXCR2 ligand expression and recruits PMN-MDSCs, thereby establishing a suppressive myeloid niche rather than a primarily PD-L1-centred escape programme [234]. Overall, current evidence indicates that A20 remodels tumour–immune interaction through multiple context-specific outputs, including checkpoint-associated signalling, loss of cytotoxic surveillance and chemokine-driven recruitment of suppressive myeloid cells [223, 225, 234, 235].

#### A20 in therapy response and resistance

##### Chemotherapy and radiotherapy

A20 is increasingly linked to response to chemotherapy and radiotherapy [226, 232, 237]. In OSCC and NPC, A20-associated control of RNF168-dependent DNA repair has direct implications for sensitivity to cisplatin and irradiation [226, 232]. The available evidence indicates that reduced or altered A20 can modify repair pathway choice and thereby influence genotoxic vulnerability [226, 237]. These findings arise from cellular perturbation studies, DNA repair assays, and therapy-response experiments, and represent some of the clearest examples in which A20 biology is directly connected to treatment outcome [232].

In NSCLC, A20 has also been linked to cisplatin sensitivity, again through DDR-associated mechanisms [238]. However, compared with head-and-neck cancers, the interpretation in lung cancer is complicated by concurrent effects of A20 on inflammatory signaling and immune escape [8, 13, 60]. A20 therefore contributes to therapy response in NSCLC, but the dominant mechanism may vary according to tumour context [13, 225, 228, 238].

##### Targeted therapy

Targeted-therapy resistance represents a second clinically important area of A20 biology [8, 60, 225, 228]. In HCC, the TNFAIP3/heat shock protein 90 (HSP90) complex has been associated with attenuation of A20-dependent suppression of inflammatory signaling and with sorafenib resistance [229]. Experimental inhibition of HSP90 restores A20-associated activity and improves treatment response, supporting a causal relationship between altered A20 function and targeted-therapy resistance in this setting [8].

In B-Raf proto-oncogene, serine/threonine kinase (BRAF)-mutant melanoma, A20 overexpression has been linked to vemurafenib resistance, largely through activation of Akt as an alternative survival pathway after BRAF inhibition [228]. This places A20 at the intersection of oncogenic kinase targeting and adaptive survival signaling, and provides a mechanistic explanation for the survival-promoting role of A20 in this disease context [8, 60, 228].

##### Immune checkpoint blockade

A20 has also emerged as a candidate determinant of response to immune checkpoint blockade (ICB) [8, 61, 172]. The strongest evidence currently comes from melanoma and lung adenocarcinoma, where A20-associated regulation of PD-L1 and CD8⁺ T-cell function has been linked to altered responsiveness to anti-PD-1/PD-L1 therapy [13, 225]. These data support the view that A20 status may influence ICB efficacy in selected tumour types, although the current literature does not justify treating A20 as a universally predictive biomarker across cancer [8, 13, 60, 228].

Taken together, the therapy-response literature supports a clinically relevant role for A20 in genotoxic sensitivity, targeted-therapy resistance, and checkpoint responsiveness [237, 238]. The mechanistic basis is most clearly defined where A20 has been directly linked to DDR control or adaptive survival signaling, and remains more variable in tumours dominated by immune-microenvironmental effects [239].

#### Representative cancer vignettes

##### Hepatocellular carcinoma

Hepatocellular carcinoma represents one of the clearest tumour-suppressive contexts for A20 [8]. A20 restricts metastatic competence through suppression of mTORC2/Akt/Rac1 signaling and limits glycolytic adaptation through PFKL-associated regulation [230]. Reduced A20 expression in HCC is therefore associated with more aggressive, metabolically reprogrammed disease. Additional evidence linking the TNFAIP3/HSP90 complex to sorafenib resistance extends this role into treatment response [229].

##### Melanoma

Melanoma provides one of the clearest tumour-promoting contexts for A20 [224]. Elevated A20 supports Akt-dependent survival, promotes invasive behaviour, increases PD-L1 expression, and weakens antitumour T-cell activity [228]. It also contributes to vemurafenib resistance and reduced efficacy of anti-PD-1 therapy. This combination of survival signaling, immune escape and treatment resistance makes melanoma a particularly strong example of context-dependent oncogenic A20 function [224, 228].

##### NSCLC

NSCLC illustrates the complexity of A20 biology in a single tumour type [225]. Reduced A20 expression is associated with aggressive clinical behaviour, yet A20 also modulates TBK1–STAT1-dependent IFN-γ sensitivity, PD-L1 induction, and CD8⁺ T-cell surveillance. In addition, DDR-related effects influence cisplatin responsiveness [13]. NSCLC therefore exemplifies a tumour in which both tumour-suppressive and immune-regulatory roles of A20 coexist [13, 225].

##### OSCC/NPC

OSCC and NPC are linked most strongly by DDR-associated A20 function [233]. In both malignancies, A20 regulates RNF168-dependent histone ubiquitination and influences sensitivity to radiotherapy and platinum-based chemotherapy [231]. These cancers therefore anchor the genome-stress and therapy-response branch of tumour-intrinsic A20 biology [232, 233].

##### Prostate cancer

In prostate cancer, the most distinctive role of A20 lies in microenvironmental remodeling [231]. Through regulation of the IKKβ/ARID1A/NF-κB axis and CXCR2 ligand expression, altered A20 signaling promotes recruitment of suppressive myeloid cells and establishes an immune-evasive niche [234]. This makes prostate cancer a particularly relevant example of A20-dependent control of the tumour microenvironment.

Across malignancies, A20 therefore does not conform to a single oncogenic or tumour-suppressive category [60]. Its clinical significance lies in the specific pathological programme with which it is coupled, including genome-stress handling, metabolic adaptation, immune escape, or treatment resistance [8] (Table 3).

### Other diseases

The disease associations of A20 extend beyond autoimmunity, cancer and infection into a broader set of disorders characterized by barrier dysfunction, maladaptive tissue remodeling, vascular injury, neuroimmune stress and immunometabolic imbalance [210, 240]. In these settings, the clinical relevance of A20 is most apparent when the dominant vulnerable compartment and the principal pathological output are clearly defined. Across epithelial tissues, reduced A20 activity is associated with barrier failure and chronic inflammatory remodeling [5]. In stromal and mesenchymal systems, altered A20 function is linked to fibrosis, matrix loss or aberrant ossification [210]. In vascular, neural and metabolic contexts, A20 most often appears at the interface between inflammatory restraint and tissue protection under stress [241].

#### Barrier and epithelial inflammatory disorders

##### Inflammatory bowel disease

Inflammatory bowel disease (IBD) is one of the clearest examples in which A20 protects a barrier tissue from chronic inflammatory injury [168]. The defining pathological feature in this setting is not inflammation alone, but the coupling of mucosal immune activation to intestinal epithelial vulnerability. A20 constrains epithelial injury by limiting inflammatory signaling and by protecting intestinal epithelial cells from death programmes that compromise barrier integrity [42]. In Tnfaip3-deficient mouse models, intestinal epithelial cells show increased sensitivity to TNFR1-mediated cell death, leading to exaggerated mucosal damage and impaired barrier function [167]. Additional mechanistic studies indicate that A20 influences the ubiquitination state of RIPK1, thereby limiting RIPK3/Caspase-8-associated death signaling in epithelial compartments [169].

Post-transcriptional repression further contributes to disease expression. In colonic epithelial cells from patients with Crohn’s disease, miR-23a targets the 3′UTR of *TNFAIP3* mRNA, suppressing its expression and increasing epithelial permeability together with inflammatory mediator release [166]. A20 also intersects with autophagy-associated barrier control through interaction with autophagy related 16 like 1 (ATG16L1), and disruption of this axis is associated with increased permeability and chronic mucosal inflammation [170]. These conclusions are supported by a combination of epithelial-cell studies, mouse models with epithelial vulnerability, and RNA-regulatory analyses. The most distinctive disease-specific role of A20 in IBD is therefore preservation of epithelial barrier competence under inflammatory stress.

##### Airway–mucosal inflammatory disorders

In airway disease, A20 is linked to epithelial restraint of persistent type 2 and remodeling-associated inflammation. This is most evident across asthma, allergic rhinitis and chronic rhinosinusitis, which share a disease architecture in which epithelial activation, barrier disturbance and chronic mucosal remodeling are tightly coupled.

In asthma, reduced A20 expression is associated with heightened sensitivity of airway epithelial cells to allergenic and inflammatory stimulation, accompanied by increased production of IL-33, thymic stromal lymphopoietin (TSLP) and interleukin-25 (IL-25), activation of group 2 innate lymphoid cell (ILC2)- and Th2-associated programmes, and progression towards structural remodeling [171, 172]. Functional studies further indicate that A20 limits airway inflammation by reducing STAT6-associated signaling and restraining chronic cytokine output. In allergic rhinitis, A20 is linked to epithelial barrier maintenance, including preservation of tight-junction-associated proteins, whereas reduced A20 is associated with increased allergen penetration and chronicity [172]. In chronic rhinosinusitis, particularly in polypoid disease, reduced A20 expression is associated with persistent type 2 inflammation and epithelial–mesenchymal transition, indicating that impaired epithelial restraint contributes directly to mucosal remodeling [15, 173, 174].

The strongest evidence across this module derives from airway epithelial studies, ovalbumin (OVA)- or LPS-based inflammatory models, and therapeutic perturbation systems in which restoration of A20-associated activity reduces inflammatory and remodeling phenotypes [175]. These findings identify the airway epithelium, rather than the inflammatory infiltrate alone, as the critical compartment through which A20 dysfunction is expressed in chronic upper and lower airway disease.

##### Periodontal inflammatory disease

Periodontal inflammatory disease represents a barrier-associated disorder at the interface of mucosal immunity and alveolar bone destruction [177]. In healthy gingiva, *TNFAIP3* is constitutively expressed, whereas inflamed tissues show reduced expression and evidence of insufficient negative feedback on inflammatory signaling [181]. In periodontal ligament cell models, microbial stimuli and nicotine enhance NLRP3/caspase-1-associated pyroptotic signaling, whereas A20 promotes autophagy and reduces pyroptosis-linked inflammatory output [177, 178]. A20 also protects gingival epithelial integrity by limiting IL-6 and IL-8 secretion and by reducing apoptosis-associated injury [180].

At the bone interface, A20 influences osteoclastogenic remodeling by reprogramming TRAF6 ubiquitination, reducing signaling compatible with osteoclast differentiation and lowering the receptor activator of nuclear factor kappa-B ligand (RANKL)/osteoprotegerin (OPG) ratio [176, 179, 181]. This is one of the more mechanistically distinctive aspects of periodontal disease in the A20 field, because it links mucosal inflammatory dysregulation to local bone loss within the same tissue niche [95]. The current evidence derives from gingival tissue analyses, periodontal ligament cell experiments, and functional studies of osteoclast-associated signaling, with human genetic support remaining less consistent [179].

##### Skin inflammatory disorders

Skin disease provides an additional epithelial context in which A20 restrains chronic inflammatory activation [182]. In atopic dermatitis, A20 suppresses NF-κB-dependent inflammatory responses and reduces expression of adhesion molecules and epithelial alarmins, thereby limiting cutaneous inflammation. Nutritional and pharmacological interventions that increase A20 expression, including zinc and formononetin, reduce inflammatory cytokine output and attenuate barrier-associated inflammation in experimental systems [184]. In these models, the disease relevance of A20 is supported mainly by keratinocyte-based studies, perturbation of upstream regulators such as G protein-coupled estrogen receptor 1 (GPER1), and inflammatory readouts rather than by cell-type–specific in vivo deletion [183].

A20 has also been implicated in other epithelial skin contexts, including acne-associated keratinocyte inflammation and advanced glycation end products (AGEs)–receptor for advanced glycation end products (RAGE)-associated pigmentation and skin aging, where reduced A20 expression is linked to NLRP3-associated inflammatory output and altered keratinocyte or fibroblast stress responses [2, 185]. These findings extend the relevance of A20 across cutaneous inflammatory states, although the mechanistic depth remains greater for barrier-associated inflammatory regulation than for pigmentation-related phenotypes.

#### Fibrotic and inflammatory remodeling across organs

##### Pulmonary fibrosis

Pulmonary fibrosis is characterized by persistent inflammatory injury followed by maladaptive fibroproliferative remodeling [187]. In this setting, the most distinctive disease-specific role of A20 is restraint of the transition from inflammatory stress to fibrotic tissue remodeling [188]. Reduced A20 activity has been linked to sustained transforming growth factor beta (TGF-β)/SMAD family member 2/3 (Smad2/3) signaling, increased myofibroblast differentiation and excessive collagen deposition [240]. In parallel, A20 protects alveolar epithelial cells from apoptosis and reduces ROS accumulation, linking inflammatory restraint to preservation of epithelial integrity [159].

Additional mechanistic studies implicate IL-33-activated interstitial macrophages, NLRP3-associated inflammatory signalling, and glycogen synthase kinase 3 beta (GSK-3β)/C/CCAAT/enhancer-binding protein beta (C/EBPβ) pathways in amplification of fibrosis when A20 function is reduced [186]. The evidence in this disease context derives primarily from fibrosis models, alveolar macrophage and epithelial studies, and pathway-focused functional analyses. Pulmonary fibrosis therefore represents an organ-remodeling state in which A20 acts mainly by limiting the conversion of chronic inflammation into stromal fibrogenesis [159, 187, 188].

##### Renal inflammatory and fibrotic disease

In renal disease, A20 is linked to preservation of tissue integrity under conditions of inflammatory, oxidative and fibrotic stress [191–193]. In renal fibrosis, the most distinctive pathway currently associated with A20 is the peroxisome proliferator-activated receptor gamma coactivator 1-alpha (PGC-1α)–*TNFAIP3* axis, in which PGC-1α supports mitochondrial fitness, increases A20 expression and reduces NLRP3-associated inflammatory activity [189]. TGF-β1 antagonizes this pathway and accelerates fibrotic remodeling, indicating that reduced A20 is functionally integrated into a broader programme of mitochondrial dysfunction and stromal injury [193].

Other renal contexts reinforce the importance of compartment specificity. In lupus nephritis, autophagy-dependent A20 degradation has been linked to RNF168 nuclear translocation, enhanced NF-κB signaling and podocyte injury [193]. In acute kidney injury, the *TNFAIP3* I325N variant has been associated with reduced tubular apoptosis and stronger antioxidant defense [3]. In diabetic and inflammatory glomerular disease, microRNA-29b (miR-29b) and exosomal microRNA-21-5p (miR-21-5p) have been reported to suppress *TNFAIP3*, thereby amplifying NF-κB signaling, ROS accumulation and inflammasome-associated injury [190]. These observations derive from renal cell studies, disease models and variant-associated functional analyses, and together support a role for A20 in renal protection across epithelial, mesangial and podocyte compartments.

##### Hepatic inflammatory and fibrotic disease

In the liver, A20 is engaged across acute injury, chronic inflammatory remodeling and metabolic liver disease [12]. The most distinctive feature of hepatic A20 biology is its sensitivity to regulation at the level of protein stability. In acute liver injury, ring finger protein 31 (RNF31) promotes A20 ubiquitination and proteasomal degradation, thereby increasing ROS accumulation and inflammatory cytokine production; conversely, RNF31 deficiency stabilizes A20 and limits tissue damage in D-galactosamine (D-Gal)/LPS-induced injury [194]. These data provide a relatively direct mechanistic link between altered A20 abundance and hepatic inflammatory injury.

Additional evidence indicates that A20 can be induced by nucleotide-binding oligomerization domain-containing protein 1 (NOD1)-associated innate signaling in a manner that reduces apoptosis and inflammation in a Kupffer cell– and TNF-dependent context [196]. In chronic liver disease, including fibrosis and non-alcoholic fatty liver disease (NAFLD)/non-alcoholic steatohepatitis (NASH), A20 has been linked to inflammatory-metabolic remodeling, although the directionality of its role is not uniform across all reports [195]. Some studies associate A20 repression with sustained NF-κB activation and inflammatory progression, whereas others link A20 to metabolic regulatory circuits that influence lipogenesis and stress adaptation [197, 199]. The strongest disease-specific conclusion is therefore that A20 participates in hepatic protection against inflammatory and oxidative injury, while its metabolic role in chronic liver disease remains more context dependent [198].

#### Bone–cartilage inflammatory remodeling

##### Osteoarthritis

In osteoarthritis, the most coherent disease-specific role currently supported for A20 is not a generalized restraint of inflammatory cartilage catabolism, but modulation of osteochondral remodeling, particularly within the subchondral bone compartment. In experimental OA, human osteoarthritic articular cartilage stem cells suppress osteoclast formation and improve abnormal subchondral bone remodeling, and these effects are strengthened by osteoarthritic synovial fluid or TNF-α and are partially mediated by secreted A20, as shown by recombinant-protein add-back, neutralization, and *TNFAIP3* gain- and loss-of-function experiments [201]. A complementary study extended this framework to the osteoblast compartment, showing that skeletal stem-cell-derived A20 limits necroptosis of subchondral osteoblasts and is associated with improved trabecular microarchitecture in rat OA [200]. Recent pharmacological work further places *TNFAIP3* within a broader miR93/TLR/NF-κB and AMPK/SIRT1-responsive network in experimental OA, but this should be interpreted as pathway-level support rather than direct evidence that endogenous A20 is a primary driver of human OA [167].

##### Intervertebral disc degeneration

In intervertebral disc degeneration, the most distinctive disease-specific feature is the coupling of A20 to mTOR-dependent autophagy and mitophagy in nucleus pulposus cells [94]. Functional studies indicate that A20 restrains mTOR activity, promotes BCL2 interacting protein 3 (BNIP3)-associated mitophagy/autophagy, and preserves extracellular matrix integrity [202]. Small interfering RNA (siRNA)-mediated inhibition of *TNFAIP3* increases mTOR signaling, suppresses autophagy and accelerates matrix loss, whereas the mTOR inhibitor Torin1 reverses these changes [63]. This combination of knockdown and pharmacological rescue provides relatively strong pathway-level evidence linking A20 to disc homeostasis under inflammatory stress.

##### Osteoporosis

Among bone-remodeling disorders, osteoporosis provides some of the strongest mechanistic evidence in the disease literature [203]. The key disease-specific output is excessive osteoclastogenesis. Osteoclast-specific deletion of *TNFAIP3* causes severe osteoporosis without overt inflammatory arthritis, demonstrating that A20 directly regulates bone remodeling independently of generalized systemic inflammation [204]. Mechanistically, A20 is recruited to the activated RANK complex, and its ZnF4/ZnF7-mediated ubiquitin-binding function appears to be more important than deubiquitinase activity alone for suppressing osteoclast differentiation [33]. This is one of the clearest examples in which cell-type–specific genetics and domain-aware mechanistic inference converge on a structurally defined disease phenotype.

##### Ankylosing spondylitis

In ankylosing spondylitis, reduced A20 has been linked to pathological new bone formation rather than to bone loss [206]. The most distinctive pathological output in this setting is therefore inflammation-associated ossification. In mesenchymal stem cell systems, reduced *TNFAIP3* is associated with enhanced Wnt family member/beta-catenin (Wnt/β-catenin) signaling and increased osteogenic differentiation. Regulatory RNA circuits, including the maternally expressed 3 (MEG3)/microRNA-125a-5p (miR-125a-5p)/*TNFAIP3* axis, further support a role for post-transcriptional A20 control in limiting aberrant ossification [205] These data derive primarily from mesenchymal cell studies and pathway-focused analyses, and support a disease-specific role for A20 at the interface of chronic inflammation and pathological bone formation.

#### Vascular and organ-protective inflammatory remodeling

##### Atherosclerosis

Atherosclerosis is a chronic inflammatory vascular disease in which A20 integrates endothelial protection with suppression of macrophage-driven plaque inflammation [55, 208]. In endothelial cells, A20 enhances endothelial nitric oxide synthase (eNOS) expression and activity through the extracellular signal-regulated kinase 5 (ERK5)–Kruppel-like factor 2 (KLF2) axis, thereby limiting TNF-induced endothelial injury [210]. In macrophages exposed to oxidized low-density lipoprotein (oxLDL), reduced A20 expression is associated with increased NF-κB activation and inflammatory cytokine production, while restoration of A20 limits inflammatory activation, foam-cell formation and plaque progression. These observations are supported by endothelial and macrophage studies, oxLDL-based functional systems and pathway perturbation analyses [207].

A20 also influences the vascular immune interface through regulation of adhesion molecules. Increased A20 expression reduces ICAM-1 and VCAM-1, thereby limiting monocyte adhesion and vascular inflammation [211]. RNA-regulatory pathways, including microRNA-23b (miR-23b) and long intergenic non-protein coding RNA 1140 (LINC01140), modulate this axis and further shape macrophage inflammatory behaviour [207, 209]. The most distinctive disease-specific role of A20 in atherosclerosis is therefore preservation of vascular homeostasis at the combined levels of endothelial integrity, macrophage activation and leukocyte recruitment.

##### Other cardiac inflammatory conditions

In other cardiac disorders, A20 appears to function primarily as a tissue-protective regulator that limits inflammatory and death-associated myocardial injury. In viral myocarditis, reduced *TNFAIP3* expression increases cardiomyocyte susceptibility to inflammatory injury and apoptosis, whereas RNA regulators such as microRNA-1a-3p (miR-1a-3p) contribute to suppression of A20 [52]. In dilated cardiomyopathy, microRNA-218-5p (miR-218-5p)–mediated *TNFAIP3* downregulation is linked to activation of TGF-β-associated remodeling and fibrosis [212]. These observations support a protective role for A20 in stressed myocardium, although the evidence is more heterogeneous and less structurally integrated than in atherosclerosis.

#### Neuroimmune, hematopoietic and immunometabolic contexts

##### Neurological disorders

In neurological disease, the most recurrent disease-specific role of A20 is preservation of the blood–brain barrier and limitation of neuroinflammatory injury. In cerebral endothelial systems, lysosomal degradation of A20 increases susceptibility to necroptosis, disrupts barrier integrity and facilitates accumulation of pathological signals such as amyloid beta (amyloid-β) [151]. In subarachnoid hemorrhage, increased A20 expression reduces early brain injury by limiting inflammatory signaling and inflammasome-associated output, whereas in ischemia–reperfusion injury mucosa-associated lymphoid tissue lymphoma translocation protein 1 (MALT1)-mediated degradation of A20 has been linked to enhanced RIPK3-associated damage [20, 214, 215]. Additional studies in microglial and neuronal systems implicate A20 in suppression of NLRP3-associated neuroinflammation [29, 151]. The available evidence derives from neurovascular and glial cell studies, injury models and pathway perturbation experiments, and supports a role for A20 in maintaining neurovascular immune restraint [213].

##### Hematological inflammatory disorders

In hematological disease, A20 is most closely linked to preservation of hematopoietic homeostasis under chronic inflammatory stress [242]. In hematopoietic stem and progenitor cells, reduced A20 expression is associated with myeloid-biased differentiation and loss of regenerative resilience during aging [216]. In myelodysplastic syndromes, lower *TNFAIP3* expression correlates with elevated inflammatory cytokines and a permissive inflammatory microenvironment [217]. Additional evidence indicates that chronic inflammatory signaling can shift hematopoietic stem-cell programs through a TLR–TRAF6–*TNFAIP3* axis, and that A20 binding inhibitor of NF-κB 1 (ABIN1)–*TNFAIP3* cooperation is required to limit type I interferon-associated cytopenic phenotypes [218]. These conclusions are supported by a combination of hematopoietic functional studies, disease-associated expression analyses and genetically modified mouse models.

##### Immunometabolic and endocrine disorders

In metabolic and endocrine disease, A20 most often appears at the interface of inflammatory regulation, oxidative stress and tissue-specific adaptation [243]. In diabetes, A20 preserves β-cell integrity by limiting cytokine-induced inflammatory and apoptotic injury, and increased A20 expression improves islet graft survival after transplantation [221]. In vascular smooth-muscle cells, A20 also restrains high-glucose-induced osteogenic conversion and calcification through a zinc/GPR39-dependent pathway that upregulates *TNFAIP3* and suppresses NF-κB signaling [219, 220].

In obesity, the role of A20 is more context dependent. In high-fat-diet models, *TNFAIP3* deficiency in bone-marrow-derived cells, particularly macrophages, has been associated with protection from obesity and insulin resistance through altered fatty-acid oxidation and mitochondrial metabolism [61]. This observation is important because it indicates that the biological effect of A20 in immunometabolic disease is not uniformly protective and depends on the compartment and metabolic state examined.

## A20-directed and A20-informed therapeutic strategies: current status and translational challenges

Translating A20 biology into therapy requires a distinction between direct A20 modulation and interventions guided by A20-regulated pathways. Direct restoration, stabilization, inhibition, gene delivery, mRNA therapy and peptide-based approaches remain largely preclinical, whereas several downstream pathways linked to A20—including cytokine signaling, immune checkpoints and regulated cell-death pathways—are already pharmacologically actionable or under clinical investigation. Because A20 can either protect tissues or support pathological cell survival depending on context, therapeutic direction must be defined by cell type, disease stage and pathway state. This section evaluates the current maturity, opportunities and limitations of both A20-directed and A20-informed strategies.

### Therapeutic rationale and current clinical status

A20 is an therapeutically attractive because it occupies a nodal position at the intersection of inflammatory restraint, tissue protection, and stress adaptation [244] (Table 4). Through these functions, A20 influences disease-relevant processes including chronic cytokine amplification, inflammatory cell death, fibrotic remodeling, antimicrobial responses, and tumour immune escape [2, 3, 127, 225]. Reduced A20 expression or function is therefore associated with autoimmune and autoinflammatory disease, fibrosis, and tissue injury, whereas increased or rewired A20 activity can support stress tolerance, immune evasion, and treatment resistance in selected cancers [13, 225].

**Table 4:** Major translational strategies and challenges in targeting A20

Strategy class	Direction of modulation	Disease contexts in which rationale is strongest	Representative evidence	Major advantage	Key limitation/risk
Direct pharmacological targeting	Directly enhance or inhibit A20 protein function	Conceptually broad, but currently limited by protein complexity	A20 contains an OTU catalytic domain and multiple ZnF modules that mediate ubiquitin recognition, complex engagement and context-dependent editing	Potentially high mechanistic specificity if domain-selective modulators can be developed	Technically difficult; multidomain structure; simple “A20 agonist” or “A20 inhibitor” may not capture full biological complexity
Pathway-oriented targeting	Target A20-linked pathways rather than A20 itself	Inflammatory, fibrotic and selected malignant settings	Intervention at TRAF6/NF-κB, inflammasome/IL-1, RIPK1-associated death pathways, profibrotic circuits, or PD-1/PD-L1-associated immune-evasive programmes	More immediately actionable and currently more advanced than direct A20 modulation	Indirect; may not accurately reflect the true functional state of A20 in specific cell compartments
A20 restoration or stabilization	Restore, stabilize or enhance A20 activity	Inflammatory disease, fibrotic disease and tissue injury	In systemic sclerosis models, A20 reduction in stromal/fibroblast compartments promotes inflammatory and fibrotic responses; restoring A20 suppresses TRAF6/NF-κB and TGF-β-associated fibrotic signaling	Mechanistically aligned with protective A20 states in inflammation and fibrosis	May be harmful if it reinforces tumour-cell survival, weakens antimicrobial defense or suppresses antitumour immunity
A20 attenuation in selected cancers	Reduce A20-associated signalling in tumour-supportive contexts	Selected tumours in which A20 promotes immune escape or treatment resistance	In colorectal cancer, A20 downregulation improves antitumour immune responses and enhances PD-1 blockade efficacy; higher A20 expression correlates with lower immune-cell infiltration and poorer outcome	May enhance antitumour immunity and improve checkpoint-blockade response in selected tumours	Not broadly applicable; risks uncontrolled inflammation or tissue injury; requires separation of tumour-suppressive from tumour-supportive A20 contexts
RNA/gene-based A20 restoration	Deliver A20 mRNA or gene-based constructs to restore A20 expression	Systemic sclerosis and tissue-restricted inflammatory injury	Lipid nanoparticle A20 mRNA restores A20 protein independently of endogenous transcription and improves skin and lung fibrosis; eye-targeted A20 gene therapy reduces inflammatory injury and pathological neovascularization	Provides the most direct route to restoring A20 when endogenous TNFAIP3 transcription is repressed	Delivery, durability, tissue specificity and off-target effects remain major barriers
A20-derived peptide approaches	Use A20-derived functional peptides to mimic protective A20 mechanisms	Acute inflammatory tissue injury, especially inflammasome-linked injury	A20-derived peptide alleviates ox-dsDNA-induced pyroptosis and improves survival by interfering with the NEK7–NLRP3 interaction	Mechanistically focused and potentially avoids full-length multidomain protein delivery	Still preclinical; uncertain stability, delivery efficiency and disease generalizability
RNA interference or knockdown approaches	Suppress A20 expression in contexts where A20 supports pathology	Tumour settings with A20-linked immune escape or treatment resistance	RNA interference and related knockdown strategies are mechanistically attractive in tumour contexts, but remain less mature than restoration approaches	Potential to weaken tumour-supportive A20 activity	Selectivity is the major limitation because broad suppression may affect tumour cells, stromal cells and immune compartments differently
Biomarker-guided stratification	Use A20-associated signatures to guide patient selection and therapeutic direction	Broadest near-term translational use across inflammatory disease, fibrosis and cancer	TNFAIP3 mRNA alone is insufficient; composite signatures integrating A20 abundance, pathway state and cellular compartment are likely needed	Clinically realistic and compatible with context-specific intervention	Requires high-resolution biomarkers; bulk tissue expression may obscure stromal, tumour-cell or immune-compartment-specific A20 states

*A20* tumor necrosis factor alpha-induced protein 3, *TNFAIP3* tumor necrosis factor alpha-induced protein 3, *OTU* ovarian tumor domain, *ZnF* zinc-finger, *TRAF6* TNF receptor-associated factor 6, *NF-κB* nuclear factor kappa B, *IL-1* interleukin-1, *RIPK1* receptor-interacting serine/threonine-protein kinase 1, *PD-1* programmed cell death protein 1, *PD-L1* programmed death-ligand 1, *TGF-β* transforming growth factor beta, *mRNA* messenger RNA, *ox-dsDNA* oxidized double-stranded DNA, *NEK7* NIMA-related kinase 7, *NLRP3* NOD-like receptor family pyrin domain-containing 3, *RNA* ribonucleic acid

However, the therapeutic maturity of these approaches should be clearly distinguished. At present, no approved drug directly and selectively activates or inhibits A20, and A20-directed mRNA, gene-delivery, peptide, and knockdown strategies remain predominantly preclinical. Available human studies involving TNFAIP3 mainly address genetic variation, disease association, biomarker potential, or natural history rather than therapeutic modulation of A20 itself. Thus, A20 should currently be regarded as an emerging translational target rather than an established drug target.

By contrast, several pathways functionally connected to A20 are already pharmacologically actionable. Cytokine-directed therapies and immune-checkpoint inhibitors are established in selected inflammatory diseases and cancers, whereas inhibitors of RIPK1 and other A20-linked signaling nodes have entered early-phase clinical development. These treatments should be described as A20-informed or pathway-oriented interventions rather than direct A20-targeted therapies, because their clinical effects do not depend on selective modulation of A20 protein function.

The same biological centrality that makes A20 therapeutically attractive also constrains its translation. A20 is not a pathway-restricted effector but a context-dependent checkpoint whose consequences vary according to cell type, disease stage, subcellular localization, and the surrounding inflammatory or metabolic environment [10, 11, 127]. A20 restoration may be protective in inflammatory and fibrotic disorders [3, 188, 236, 244], whereas persistent A20 activity may promote tumor-cell survival or immune escape in selected malignancies [13, 223, 225]. Therapeutic direction must therefore be defined for each disease and cellular compartment rather than inferred from A20 abundance alone.

### Direct A20 modulation versus A20-informed pathway targeting

Direct pharmacological modulation of A20 remains conceptually attractive but technically challenging. A20 contains an N-terminal ovarian tumor domain and multiple C-terminal zinc-finger modules that mediate deubiquitination, ubiquitin recognition, protein-complex recruitment, and context-dependent ubiquitin editing [245, 246]. These structurally and functionally distinct domains provide several possible intervention points, but they also make a simple “A20 agonist” or “A20 inhibitor” model biologically inadequate. Modulating one domain may not reproduce the effects of changing full-length A20 expression and may produce different outcomes across receptor complexes or cell types [40, 42, 245].

Consequently, pathway-oriented intervention is currently more advanced than direct A20 modulation. In inflammatory and fibrotic disease, potentially actionable A20-linked nodes include TRAF6/NF-κB signaling, inflammasome/IL-1 pathways, RIPK1-associated inflammatory cell death, and TGF-β-associated profibrotic circuits [3, 188, 247, 248] In cancer, relevant interventions include blockade of PD-1/PD-L1-associated immune-evasive programs and targeting of stress-survival or DNA-damage-response pathways influenced by A20 [223, 249].

Nevertheless, clinical activity against an A20-linked pathway should not be interpreted as evidence that A20 itself has been therapeutically validated. These pathway-directed agents act on broader signaling networks and may remain effective regardless of whether A20 dysregulation is the dominant disease mechanism. Demonstrating that A20 status predicts treatment response will therefore require prospective biomarker studies rather than mechanistic inference alone.

### Preclinical A20-restoration and attenuation strategies

In inflammatory and fibrotic disease, the dominant translational logic is usually restoration, stabilization, or enhancement of A20 function [2]. Recent preclinical work in systemic sclerosis illustrates this clearly [10]. In patient-derived tissues and fibroblast systems, A20 is reduced whereas its repressor downstream regulatory element antagonist modulator (DREAM) is increased, and both global and fibroblast-specific loss of A20 exaggerate inflammatory and fibrotic responses in skin and lung [188]. These observations provide cell-intrinsic evidence that insufficient A20 in stromal compartments can directly promote fibrosis.

The same logic is reinforced by more explicitly therapeutic studies. A20 mRNA packaged in lipid nanoparticles restored A20 expression, suppressed TRAF6/NF-κB signaling, reduced TGF-β-associated fibrotic signaling [5, 250], and attenuated skin and lung fibrosis in preclinical systemic sclerosis models. In a separate acute kidney injury study, an A20-derived peptide alleviated oxidized double-stranded DNA (ox-dsDNA)-induced pyroptosis and improved survival in mice, with mechanistic data linking A20 to competitive interference with the never in mitosis gene A-related kinase 7 (NEK7)–NLRP3 interaction [3]. Tissue-restricted gene delivery has also shown preclinical activity in ischemic proliferative retinopathy, in which ocular A20 gene therapy reduced inflammatory injury and pathological neovascularization [251].

These findings demonstrate proof of concept but do not yet establish clinical efficacy or safety. The studies remain dependent on experimental models, and key issues—including pharmacokinetics, immunogenicity, durability, dose control, tissue distribution, and manufacturing feasibility—have not been resolved in humans.

In cancer, therapeutic direction is less uniform. Recent work in colorectal cancer showed that A20 downregulation improved antitumour immune responses in vitro and enhanced the efficacy of PD-1 blockade in vivo, with higher A20 expression correlating with lower immune-cell infiltration and poorer outcome [236]. These findings do not imply that A20 should be inhibited broadly across cancer, but they do show that, in selected tumours, A20-linked signaling can be functionally aligned with immune escape rather than tissue protection [252]. Therapeutic manipulation in oncology therefore requires explicit separation of tumour-suppressive from tumour-supportive A20 contexts [253].

### Clinically actionable A20-linked pathways

Although direct A20-targeted treatment has not entered routine clinical practice, several therapeutic classes acting on A20-linked pathways are already clinically relevant. Cytokine-directed therapies targeting TNF or IL-1 are established treatments for selected immune-mediated and autoinflammatory diseases. Their clinical efficacy supports the therapeutic relevance of inflammatory networks regulated by A20, but these agents neither restore defective A20 function nor reproduce the full range of its ubiquitin-editing activities.

In oncology, PD-1 and PD-L1 inhibitors are established treatments for multiple tumor types. Experimental evidence that A20 can influence PD-L1 expression, T-cell surveillance, or checkpoint-blockade sensitivity provides a rationale for evaluating A20 as a response biomarker or resistance modifier. However, checkpoint inhibitors should not be classified as A20-targeted treatments, because their clinical use is currently guided by tumor type, stage, PD-L1 status, and other established biomarkers rather than A20 expression.

RIPK1 inhibitors represent a more direct example of an A20-linked pathway entering clinical development. Several small-molecule RIPK1 inhibitors have been evaluated in early-phase studies involving psoriasis, rheumatoid arthritis, inflammatory bowel disease, cutaneous lupus erythematosus, multiple sclerosis, and other inflammatory or neurological conditions. Clinical results have been variable, and these agents remain investigational rather than established standards of care. Their development nevertheless illustrates how mechanistic knowledge of A20-regulated cell-death pathways can identify pharmacologically accessible downstream targets.

Accordingly, the present clinical relevance of A20 biology lies primarily in pathway interpretation, biomarker development, and patient stratification rather than in direct therapeutic manipulation of A20.

### Biomarker, delivery and context-specific challenges

The principal challenge is not simply whether A20 should be increased or decreased, but where, when, and in which cellular compartment modulation should occur. Restoration may be beneficial in fibroblasts, epithelial cells, or selected immune populations during inflammatory and fibrotic disease, but the same intervention may reinforce tumor-cell survival, weaken antimicrobial defense, or suppress protective antitumor immunity [4, 5]. Conversely, reducing A20-associated signaling may enhance antitumor responses in selected cancers while increasing inflammatory or tissue-injury risks elsewhere [6].

Disease stage adds another layer of complexity. Augmenting A20 during early inflammatory or fibrotic disease may limit escalation and irreversible remodeling. In established tumors exposed to immune or therapeutic pressure, however, A20-linked stress adaptation may already be incorporated into the malignant program [253]. A20-directed interventions must therefore account for disease timing, tissue context, prior therapy, and the state of the local immune microenvironment.

Biomarker resolution is equally important. TNFAIP3 mRNA abundance alone is unlikely to represent the functional state of A20 because its activity is also influenced by protein stability, localization, post-translational modification, ubiquitin-chain recognition, and receptor-specific complex assembly [119, 188]. Composite biomarkers integrating A20 protein abundance, functional pathway activity, cell identity, and disease context are therefore likely to be more informative than bulk-tissue transcriptional measurements [44].

Finally, delivery remains a major determinant of feasibility. Systemic A20 augmentation or inhibition may produce opposing effects across stromal, epithelial, immune, and malignant compartments. Lipid nanoparticles, tissue-restricted vectors, targeted peptides, and cell-selective RNA delivery may reduce this problem, but none has yet established sufficient clinical safety, durability, and specificity for direct A20 modulation. Future translation will require not only a therapeutic modality but also a validated biomarker framework that identifies the cellular compartment, direction, and timing of intervention.

## Conclusion and future directions

A20 has emerged as a central ubiquitin-editing checkpoint that links inflammatory signal termination to immune homeostasis, tissue integrity, and stress adaptation. Across the evidence summarized in this Review, A20 is most convincingly positioned at receptor-proximal signaling interfaces and within a limited set of recurrent biological outputs, including inflammatory amplification, cell-death control, barrier protection, fibrotic remodeling, and metabolic adaptation. This functional breadth explains why A20 dysregulation is associated with such a wide spectrum of disease, ranging from monogenic autoinflammation and autoimmune disorders to chronic tissue remodeling, infection, and cancer. At the same time, the available literature indicates that A20 cannot be adequately described as a generic anti-inflammatory factor. Its biological significance lies in determining how inflammatory and stress-associated signaling are translated into disease-specific outcomes within distinct cellular and tissue compartments.

A consistent conclusion across these disease settings is that the effect of A20 is fundamentally context dependent. In autoimmune and inflammatory disorders, reduced A20 expression or function most often lowers the threshold for persistent immune activation, destabilizes tolerance, and permits chronic tissue-damaging inflammation. In barrier and stromal tissues, the same loss of restraint is expressed as epithelial injury, fibrosis, or structural remodeling. In infection, A20 shapes the balance between antimicrobial defense and host tissue protection. In cancer, its role becomes especially conditional: A20 may suppress inflammation-associated tumorigenesis in one setting, while in another it may support tumour-cell survival, immune escape, or treatment resistance. These apparently divergent effects are more coherently understood as consequences of differences in cellular compartment, local signaling architecture, ubiquitin-complex topology, and tissue stress state than as fundamentally contradictory properties of the protein itself.

Several major gaps remain. First, the rules governing substrate selection are still incompletely defined across disease systems. Although receptor-proximal NF-κB regulation is supported by comparatively strong biochemical, genetic, and domain-aware evidence, many additional functions of A20 remain defined primarily through pathway perturbation, disease-model association, or transcript-level regulation. Second, the relative contributions of the OTU catalytic domain and the ZnF-dependent ubiquitin-binding and complex-engagement modules remain unevenly resolved across tissues and pathological contexts. Third, the quality of evidence remains heterogeneous: some conclusions are anchored by human genetics, cell-type–specific in vivo models, and mechanistic studies, whereas others continue to rely mainly on expression profiling, RNA-regulatory inference, or broader functional correlation. Finally, compartment specificity remains a central challenge. In several diseases, A20 appears protective in one cellular context and potentially maladaptive in another, limiting the interpretability of bulk-tissue observations and complicating therapeutic translation.

Future work will need to define more precisely how A20 is recruited to specific signaling complexes, how domain-specific functions are deployed in different tissues, and which disease phenotypes depend on direct substrate-level regulation rather than on secondary rewiring of broader inflammatory networks. Progress in these areas will require tighter integration of domain-resolved genetics, chain-type–aware biochemical analysis, and spatially or single-cell–resolved studies with human disease data. From a translational perspective, the most plausible path forward is not indiscriminate manipulation of A20, but context-specific intervention: restoration of A20 function in selected inflammatory or fibrotic disorders, restriction of A20-linked stress adaptation in defined tumour settings, and biomarker-guided targeting of A20-associated signaling states. In this sense, the major task ahead is no longer simply to document additional disease associations, but to establish the mechanistic rules that determine when A20 acts as a protective brake, when it becomes permissive for pathology, and how that knowledge can be translated into more precise therapeutic strategies.

## Data Availability

No datasets were generated or analysed during the current study. All data and information discussed in this review are derived from previously published studies.

## Abbreviations

*TNFAIP3*: Tumor necrosis factor alpha-induced protein 3
OTU: Ovarian tumor domain
ZnF: Zinc finger
K63-linked: Lysine 63-linked ubiquitin chain
M1-linked: Methionine 1-linked (linear) ubiquitin chain
NF-κB: Nuclear factor kappa B
RIPK1: Receptor-interacting serine/threonine-protein kinase 1
TNFR1: Tumor necrosis factor receptor 1
TNF: Tumor necrosis factor
TLR: Toll-like receptor
IL-1R: Interleukin-1 receptor
MyD88: Myeloid differentiation primary response 88
IRAK: Interleukin-1 receptor-associated kinase
TRAF6: TNF receptor-associated factor 6
IL-17R: Interleukin-17 receptor
MAPK: Mitogen-activated protein kinase
JNK: C-Jun N-terminal kinase
LPS: Lipopolysaccharide
3′UTR: 3′ Untranslated region
JAK–STAT: Janus kinase–signal transducer and activator of transcription
PI3K–AKT–mTOR: Phosphoinositide 3-kinase–protein kinase B–mechanistic target of rapamycin
ILC2: Group 2 innate lymphoid cell
IFN-γ: Interferon gamma
DSS: Dextran sulfate sodium
NEK7: NIMA-related kinase 7
NLRP3: NOD-like receptor family pyrin domain-containing 3
RIPK3: Receptor-interacting serine/threonine-protein kinase 3
MLKL: Mixed lineage kinase domain-like protein
CD4: Cluster of differentiation 4
Treg: Regulatory T cell
TNFR2: Tumor necrosis factor receptor 2
TCR: T-cell receptor
BCR: B-cell receptor
HA20: Haploinsufficiency of A20
TNF-α: Tumor necrosis factor alpha
CXCL9: C-X-C motif chemokine ligand 9
CXCL10: C-X-C motif chemokine ligand 10
Th1: T helper 1
RA: Rheumatoid arthritis
BD: Behçet’s disease
SLE: Systemic lupus erythematosus
MS: Multiple sclerosis
CNS: Central nervous system
EAE: Experimental autoimmune encephalomyelitis
ICOSL: Inducible T-cell co-stimulator ligand
ICAM-1: Intercellular adhesion molecule 1
VCAM-1: Vascular cell adhesion molecule 1
HBV: Hepatitis B virus
HCV: Hepatitis C virus
HRSV: Human respiratory syncytial virus
HCoV-229E: Human coronavirus 229E
IKKβ: IκB kinase beta
NEMO: NF-κB essential modulator
IRF3: Interferon regulatory factor 3
ALV-A: Avian leukosis virus subgroup A
CLR: C-type lectin receptor
IL-10: Interleukin-10
PCB: Polychlorinated biphenyl
SOCS1: Suppressor of cytokine signaling 1
IRAK3: Interleukin-1 receptor-associated kinase 3
PD-1: Programmed cell death protein 1
DDR: DNA damage response
HCC: Hepatocellular carcinoma
HSP90: Heat shock protein 90
BRAF: B-Raf proto-oncogene, serine/threonine kinase
ICB: Immune checkpoint blockade
PD-L1: Programmed death-ligand 1
OSCC: Oral squamous cell carcinoma
NPC: Nasopharyngeal carcinoma
NSCLC: Non-small cell lung cancer
IBD: Inflammatory bowel disease
ATG16L1: Autophagy related 16 like 1
TSLP: Thymic stromal lymphopoietin
IL-25: Interleukin-25
OVA: Ovalbumin
RANKL: Receptor activator of nuclear factor kappa-B ligand
OPG: Osteoprotegerin
GPER1: G protein-coupled estrogen receptor 1
AGEs: Advanced glycation end products
RAGE: Receptor for advanced glycation end products
TGF-β: Transforming growth factor beta
Smad2/3: SMAD family member 2/3
GSK-3β: Glycogen synthase kinase 3 beta
C/EBPβ: CCAAT/enhancer-binding protein beta
PGC-1α: Peroxisome proliferator-activated receptor gamma coactivator 1-alpha
miR-29b: MicroRNA-29b
miR-21-5p: MicroRNA-21-5p
RNF31: Ring finger protein 31
D-Gal: D-galactosamine
NOD1: Nucleotide-binding oligomerization domain-containing protein 1
NAFLD: Non-alcoholic fatty liver disease
NASH: Non-alcoholic steatohepatitis
OA: Osteoarthritis
BNIP3: BCL2 interacting protein 3
siRNA: Small interfering RNA
Wnt/β-catenin: Wnt family member/beta-catenin
MEG3: Maternally expressed 3
miR-125a-5p: MicroRNA-125a-5p
eNOS: Endothelial nitric oxide synthase
ERK5: Extracellular signal-regulated kinase 5
KLF2: Kruppel-like factor 2
oxLDL: Oxidized low-density lipoprotein
miR-23b: MicroRNA-23b
LINC01140: Long intergenic non-protein coding RNA 1140
miR-1a-3p: MicroRNA-1a-3p
miR-218-5p: MicroRNA-218-5p
MALT1: Mucosa-associated lymphoid tissue lymphoma translocation protein 1
ABIN1: A20-binding inhibitor of NF-κB 1
DREAM: Downstream regulatory element antagonist modulator
ox-dsDNA: Oxidized double-stranded DNA
